# Convenient Solid-Phase Attachment of Small-Molecule Ligands to Oligonucleotides via a Biodegradable Acid-Labile P-N-Bond

**DOI:** 10.3390/molecules28041904

**Published:** 2023-02-16

**Authors:** Nadezhda O. Kropacheva, Arseniy A. Golyshkin, Mariya A. Vorobyeva, Mariya I. Meschaninova

**Affiliations:** 1 Institute of Chemical Biology and Fundamental Medicine, Siberian Branch, Russian Academy of Sciences, Novosibirsk 630090, Russia; 2 Department of Natural Sciences, Novosibirsk State University, Novosibirsk 630090, Russia

**Keywords:** 5′-functionalization, small molecules, conjugates of oligonucleotides, solid-phase synthesis, pH-sensitive phosphoramidate linkage, siRNA

## Abstract

One of the key problems in the design of therapeutic and diagnostic oligonucleotides is the attachment of small-molecule ligands for targeted deliveries in such a manner that provides the controlled release of the oligonucleotide at a certain moment. Here, we propose a novel, convenient approach for attaching ligands to the 5′-end of the oligonucleotide via biodegradable, acid-labile phosphoramide linkage. The method includes the activation of the 5′-terminal phosphate of the fully protected, support-bound oligonucleotide, followed by interaction with a ligand bearing the primary amino group. This technique is simple to perform, allows for forcing the reaction to completion by adding excess soluble reactant, eliminates the problem of the limited solubility of reagents, and affords the possibility of using different solvents, including water/organic media. We demonstrated the advantages of this approach by synthesizing and characterizing a wide variety of oligonucleotide 5′-conjugates with different ligands, such as cholesterol, aliphatic oleylamine, and *p*-anisic acid. The developed method suits different types of oligonucleotides (deoxyribo-, 2′-O-methylribo-, ribo-, and others).

## 1. Introduction

Functional nucleic acids (FNAs) (catalytic NAs, aptamers, small, interfering RNAs, antisense oligonucleotides, etc.) and FNA-based constructs represent very promising, highly selective research tools for molecular biology, as well as potential therapeutic agents for viral, oncological, and other diseases [1,2,3,4,5,6]. However, their applications for targeting certain biomolecules inside the cell face the problem of insufficient cell delivery. The negative charge of the sugar-phosphate backbone hinders NA penetration through the negatively charged cell membrane. The cell delivery of FNAs can be improved by conjugation with small, transporting molecules, such as vitamins, dendrimers, lipophilic compounds, peptides, cationic lipids, polymers, etc. (e.g., [7,8,9,10,11,12,13,14]).

Small molecules can be attached to functional NAs either noncovalently or covalently via chemical bonds, which, in turn, can be stable or labile under intracellular conditions. The use of biodegradable bonds allows for the release of an FNA cargo from the carrier molecule under the appropriate intracellular conditions, which, in turn, can enhance its biological activity. In particular, acid-labile linkers are widely used to address this issue: acetals, ketals, beta-thiopropionates, oximes, orthoesters, hydrazones, etc. [15,16,17]. In our work, we chose a phosphoramidate bond for creating an acid-labile linker. 

The literature describes several synthetic approaches to the synthesis of the conjugates of nucleotides or oligonucleotides containing a P-N-bond at the terminal or internucleoside phosphates. One of the most common and frequently used options for obtaining a phosphoramide bond is the Atherton–Todd reaction: the conversion of a dialkyl phosphite into dialkyl chlorophosphate in the presence of carbon tetrachloride, followed by the reaction with a primary amine [18,19]. Alternatively, dialkyl phosphite can be oxidized in the presence of elemental iodine, followed by interaction with amino-containing ligands [20,21]. These reactions allow for the introduction of modification during solid-phase oligonucleotide synthesis. The Staudinger reaction provides another option for obtaining such modifications by solid-phase synthesis. For example, the authors of [22,23] obtained a phosphoramide bond through the Staudinger reaction between azidoalkyl-modified lipophilic molecules and an internucleoside 2-cyanoethylphosphite of the polymer-bound protected oligonucleotide, which is formed at the condensation stage in a standard automated synthesis. Dovydenko et al. [24] proposed a variant of the solid-phase phosphotriester approach: the active 5′-arylphosphodiester derivative reacted with the polymer-bound protected oligonucleotide, with the subsequent replacement of the azole moiety by an alkyl amine. 

Otherwise, the ligands can be introduced through the P-N-bond at the 5′-/3′-end or the 2′-position of the fully deblocked oligonucleotide by activating the phosphoric acid residue with carbodiimide (EDC) in the presence of methylimidazole or with the ox/red pair triphenylphosphine/dipyridyl disulfide (PPh_3_/(PyS)_2_) [25,26,27,28,29,30,31]. Based on the last option, we proposed a synthetic approach to the 5′-modification of oligonucleotides. Here, we combine the solid-phase approach, with all its advantages, with the activation of the terminal phosphate of the protected polymer-bound oligonucleotide (ribo-, deoxyribo- or 2′-*O*-methylribo-) via PPh_3_/(PyS)_2_/DMAP, followed by the interaction with amino-containing ligands of various chemical nature.

## 2. Results and Discussion

As we mentioned above, there are two strategies for the synthesis of oligonucleotide conjugates with small molecules: (1) the introduction of ligands into a fully deblocked oligonucleotide (synthesis “in solution”), and (2) the interaction of ligands with a support-bound protected oligonucleotide (solid-phase conjugation) [32,33,34,35,36]. Each of them has its own advantages and shortcomings. From our point of view, the solid-phase approach is preferable for obtaining conjugates with small transporting molecules. Protected oligonucleotide is covalently bound with the carrier, which allows for using almost any combination of solvents, including water-organic media. This possibility becomes especially important for attaching lipophilic molecules since they require nonpolar solvents that are poorly suited to deblocked oligonucleotides. The presence of standard protecting groups in the oligonucleotide makes it possible to introduce the ligand selectively at a given position, using it in significant excesses when necessary. The easy removal of unreacted components and side products by simple washing of the carrier and high conjugation efficiency make the solid-phase approach handy and versatile.

**Figure 1:**
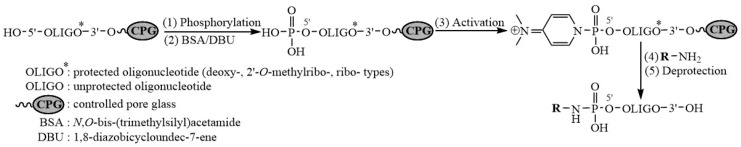
The common scheme for oligonucleotide solid-phase 5′-functionalization. R–NH_2_ structures: see Supplementary Table S1.

The common scheme of the proposed solid-phase 5′-functionalization of oligonucleotides (deoxyribo-, ribo-, and 2′-O-methylribo-) includes four steps (Figure 1): (1) introduction of a phosphate group at the 5′-end of the oligonucleotide chain during an automatic synthesis, (2) the deprotection of the terminal phosphate, (3) the activation of 5′-phosphate of the protected support-bound oligonucleotide by PPh_3_/(PyS)_2_, with the formation of the 5′-DMAP-intermediate, (4) the interaction of the intermediate with amino-modified molecules, and (5) the standard deprotection of the subsequent oligonucleotide conjugates. The approach is easy to use, does not require changes in the standard automatic protocol for the synthesis of oligonucleotides, and allows for the obtainment of a set of different conjugates, starting from one oligonucleotide. 

Our study includes three steps: the synthesis of amino-containing ligands, the development of a method for the synthesis of oligonucleotide conjugates with an acid-labile bond, and the study of the stability of an acid-labile bond at different pH values. 

### 2.1. Synthesis of Amino-containing Ligands

In order to demonstrate the possibilities of the developed approach, we used a set of small molecules bearing a primary amino group: commercially available amino-containing ligands (pyrenemethylamine, aliphatic diamine, oleylamine, propargylamine, 3-aminopropan-1-ol,), as well as home-made amino-modified cholesterol and *p*-anisic acid. Some of these molecules were reported to be used for cell delivery. In particular, lipophilic oleylamine and cholesterol are capable of interacting with cell membranes [6,7,8], and anisamide (a derivative of *p*-anisic acid) possess a high affinity for the sigma receptor 1 (δ1), which is overexpressed on the surface of cancer cells [7,8,9,37,38,39]. It is important to note that some small molecule ligands can be sensitive to the conditions of oligonucleotides deprotection. Therefore, one should carefully choose the small molecules for solid-phase conjugation with oligonucleotides and take into account the possibility of their destruction under the conditions of the final release of the oligonucleotide conjugates.

**Figure 2:**
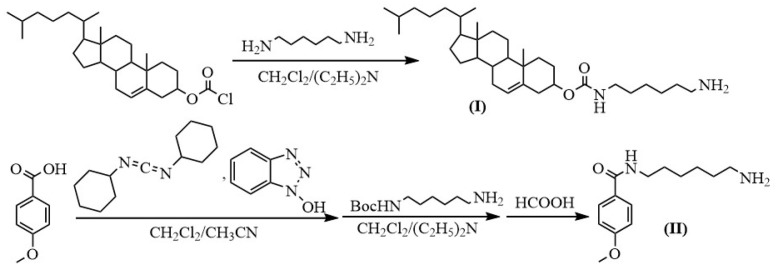
Schemes of the syntheses of amino-modified cholesterol (**I**) and *N*-(6-aminohexyl)-4-methoxybenzamide (**II**). Boc—*tert*-butyloxycarbonyl.

The synthesis of amino-containing cholesterols (**I**) was carried out according to our previous work [40] (Figure 2): 1,6-diaminohexane was used in excess to introduce cholesteryl chloroformate into the process. The carboxylic group of *p*-anisic acid was activated using *N,N*′-dicyclohexylcarbodiimide (DCC) in the presence of *N*-hydroxybenzotriazole (HOBt), analogous with [41]. The activated *p*-anisic acid reacted with the mono-*N*-Boc-protected 1,6-diaminohexane, then the Boc-protected group was removed with a formic acid, as described in [42] (Figure 2). NMR was used to confirm the structures of the ligands with amino modifications. For further processing, see Section 3: Materials and Methods.

### 2.2. Solid-Phase Synthesis of Oligonucleotide Conjugates

**Figure 3:**
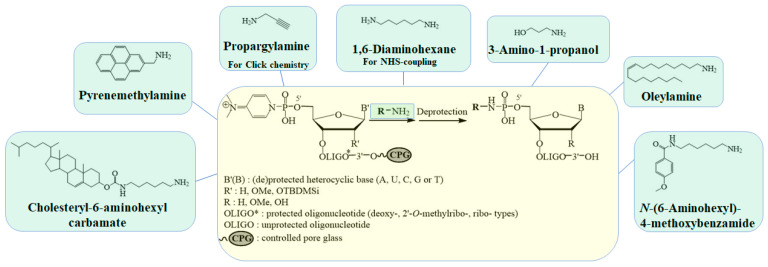
Solid-phase synthesis of 5′-conjugates of oligonucleotides containing an acid-labile P-N-bond (Table 1).

We optimized the methodology of the proposed solid-phase approach to the synthesis of 5′-modified oligonucleotides and tested its possibilities by obtaining various conjugates of the model oligodeoxyribonucleotide dT_7_ (**1–9**) (Figure 3, Table 1). 

**Figure 4:**
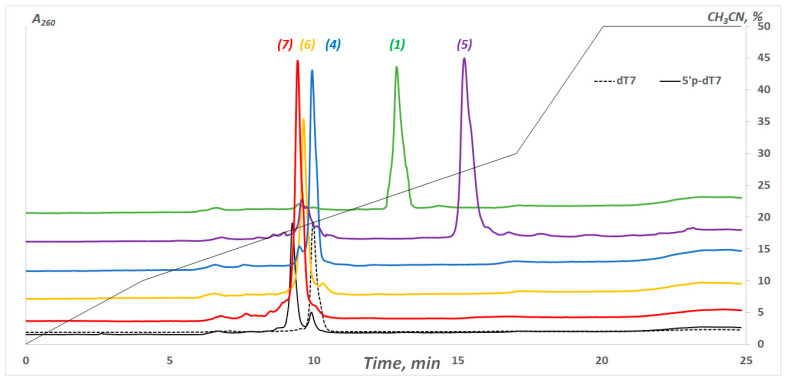
RP-HPLC analyses of reaction mixtures upon the conjugation of 5′-p-dT_7_ with different amino ligands (Table 1). dT_7_: 5′-d(TTTTTTT); 5′-p-dT_7_: 5′-p-d(TTTTTTT); (**1**): *MB*-**L_6_**-NH-p-dT_7_; (**4**): CH≡C-CH_2_-NH-p-dT_7_; (**5**): *Pyr*-CH_2_-NH-p-dT_7_; (**6**): HO-(CH_2_)_3_-NH-p-dT_7_; (**7**): NH_2_-(CH_2_)_6_-NH-p-dT_7_; *MB*-**L_6_**-NH-p-, *N*-(6-aminohexyl)-4-methoxybenzamide residue; *Pyr*-CH_2_-NH–, pyrenemethylamine residue; -p-, -P(O)(OH)-; d(N), deoxyribonucleotide. See Section 3: Materials and Methods for details.

The approach was developed on the basis of the solution method described in [30,31]. After phosphorylation in an automatic mode, this was followed by the removal of the protective groups from the phosphates of the oligonucleotide. We processed a protected polymer-bound model oligonucleotide 5′-p-dT_7_ with *N,O*-bis(trimethylsilyl)acetamide (BSA), with subsequent treatment via 1,8-diazabicyclo[5.4.0]undec-7-ene (DBU), analogous with [31] (Figure 1). It was shown that BSA provides a rearrangement of the linkage between the oligonucleotide and the support, which makes it stable. In turn, DBU gently removes the cyanoethyl-protective groups from the phosphates, leaving the oligonucleotide attached to the support [43]. Since the terminal phosphate is capable of incorporating two ligands, as was shown by the example of pyrene derivatives in [30], we additionally optimized the conditions for obtaining mono conjugates. We thoroughly selected the best solvent for each amino ligand’s solid-phase addition (Supplementary Table S1). We also showed that the formation of mono conjugates requires the removal of activating agents (Ph_3_P/(PyS)_2_/DMAP) and the three-fold washing of the support with the attached oligonucleotide before coupling with the amino-containing ligand. The treatment by an aqueous solution of methylamine or its mixture with ammonia to remove the protective groups and cleave the oligonucleotide from the polymer carrier did not destroy the P-N-bond [19,20,24]. In our work, we used an aqueous solution of methylamine. During the analyses of the reaction mixtures of the oligonucleotide conjugates after deprotection, we found an unknown product in some cases and assumed it to be the 5′-p-dT_7_ derivative, with a methylamine at the 5′-phosphate. In order to check this hypothesis, the support-bound 5′-p-dT_7_ with an activated 5′-phosphate was washed with DMSO and immediately treated with a methylamine solution. In this case, the deblocking and cleavage of the oligonucleotide from the support occur simultaneously, with methylamine attachment at the terminal phosphate (Supplementary Figure S1). The reaction mixtures were analyzed by reversed-phase high-performance liquid chromatography (RP-HPLC) and analytical gel electrophoresis (Supplementary Figure S1). The degree of conversion of 5′-p-dT_7_ into the corresponding conjugates was 75–95%, according to RP-HPLC data. Figure 4 shows some of the typical examples of chromatogram profiles for conjugates (**1, 4–7**). The nature of the ligand affected the retention time of the conjugate during RP-HPLC: lipophilic conjugates with cholesterol (**2**) and oleylamine (**3**) had the highest retention times (Table 1).

The developed approach was tested for obtaining RNA and 2′-*O*-Me-RNA oligonucleotides. As a biologically active synthetic RNA, we chose the siRNA directed to the 557–577 region of mRNA to the MDR1 gene (multiple drug resistance gene). MDR1 encodes the membrane protein P-glycoprotein that is responsible for the transmembrane efflux of such substances as lipids, steroids, peptides, bilirubin, etc., thereby providing the effect of drug resistance [44]. Modifications at the 5′-end of the sense strand of siRNA do not affect the activity of siRNA [44]. It has been shown that the use of siRNAs with the sense strand divided into two fragments is a promising approach to gene silencing [45]. Therefore, our next task was to synthesize the 5′-mono-conjugates of the RNA sense strand and the 5′-mono-conjugates of the 2′-*O*-Me RNA half of the sense strand. The necessity of 2′-*O*-protective groups during the RNA chemical synthesis adds one more deblocking step to their removal and requires the optimization of the conjugation protocol. We found that the treatment of the conjugates containing a phosphoramide bond with a standard mixture of NMP/TEA·3HF/TEA for the removal of the 2′-*O*-TBDMS protective groups partly cleaved this bond, giving the 5′-phosphate-containing oligonucleotide. However, the treatment with a 1 M solution of tetrabutylammonium fluoride in tetrahydrofuran, followed by neutralization with triethylammonium acetate buffer (pH 7.0), and desalting on a C-18 cartridge, preserves the integrity of the P-N-bond. Thus, for the complete deblocking of conjugates of various types of oligonucleotides containing an acid-labile P-N-bond, methylamine treatment suits well, with or without the TBAF treatment and desalting.

The presence of 5′-amino- or 5′-alkyne groups within the oligonucleotide allows their further functionalization via well-known reactions with NHS-activated esters or click-chemistry. We demonstrated these possibilities by the interaction of model aliphatic amino-modified oligonucleotide (**7**) or propargylamine-modified oligonucleotides (**12, 17**) with Biotin-NHS or GalNAc/FAM azide, respectively. The degree of conversion was about 80%, according to the HPLC data (Supplementary Figures S2 and S3, respectively).

All obtained oligonucleotide conjugates were isolated by preparative gel electrophoresis. The yields of the conjugates related to the first support-bound nucleoside were 18–24% (Table 1), which is comparable to the yields of nonconjugated (parent) oligonucleotides of the same length. The homogeneities of the resulting conjugates were confirmed by analytical denaturing PAGE, followed by mass spectrometry. Notably, in some cases, we observed the cleavage of the P-N-bond during mass analysis, especially when recording MALDI spectra with the use of acidic matrices (Supplementary Table S2), similar to the effect that was registered earlier for the oligonucleotide conjugates with acid-labile hydrazone linkage [46].

### 2.3. Stability of the P-N-Bond within the Oligonucleotide Conjugates at Different pH Values

When small transporting molecules are introduced into the conjugate through a labile bond, upon fulfilling their role, they have to be cleaved from the cargo in the endosomes and lysosomes and leave the therapeutic NA free to perform its function. Therefore, it is important to quantitatively assess the liability of the linkage between the oligo and the transporting ligand. For example, it has been shown that the phosphoramide bond between the oligonucleotide and polyethylene glycol was completely cleaved at 37 °C for 5 h at pH 4.7 [25]. The authors of [47] systematically studied the uptake of lipid nanoparticles loaded with siRNAs and their intracellular transport and endosomal release and found that in the course of these processes, the pH values varied in the range of 4.5–6.5, and the total time was approx. 5 h.

**Figure 5:**
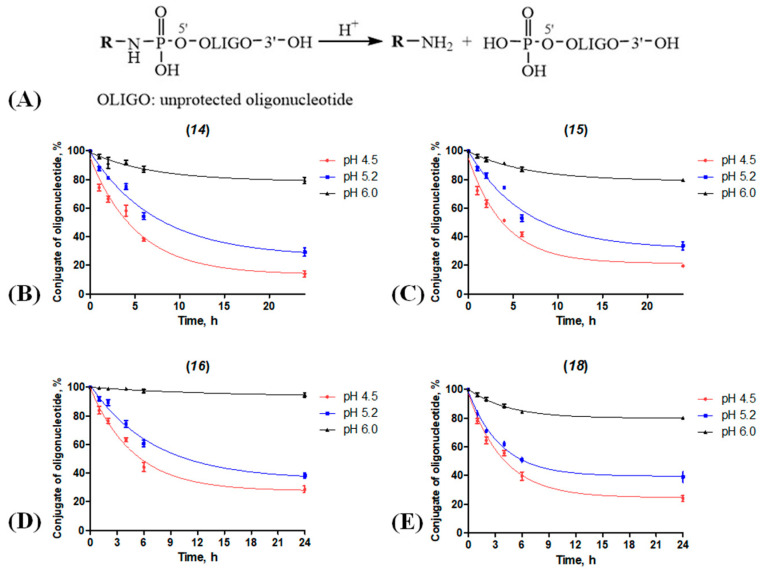
Scheme of hydrolysis (**A**) and kinetic curves of P-N-bond cleavage in conjugates of 5′-p-siDmS (**14–16, 18**, Table 1) with *p*-anisic acid (**B**), cholesterol (**C**), oleyl (**D**) and GalNAc (**E**) at different pH values. Quantification of the full-size conjugate (%, axis Y) in relation to incubation time and pH. The results are presented as mean values (±SD) from three independent experiments. Section 3: Materials and Methods for details.

We studied the stability of the phosphoramide bond within the synthesized conjugates at different pH values. The conjugates of oligonucleotide 5′-p-siDmS with *N*-(6-aminohexyl)-4-methoxybenzamide, cholesteryl-6-aminohexylcarbamate, oleylamine, and GalNAc (**14–16, 18**) were incubated in acetate buffer with pH values of 6.0, 5.2, and 4.5 at 37 °C for 1–24 h, and were then analyzed by the gel electrophoresis. According to the obtained data (Figure 5), at pH 6.0, the P-N-bond is hydrolyzed by no more than 20%, and at pH values lower than 5.2, it becomes significantly less stable. We observed the highest degree of P-N-bond cleavage at pH 4.5 for the conjugate 5′-p-siDmS with the *N*-(6-aminohexyl)-4-methoxybenzamide (**14**).

## 3. Materials and Methods

### 3.1. Chemicals and Reagents

A controlled pore glass support (CPG) derivatized with 2′-*O*-methyl-A, 2′-*O*-methyl-G, deoxythymidine, 5′,*N*-protected 2′-*O*-methylribo- (A, C, G, or U), 2′-*O*-TBDMS-ribo (A, C, G, or U) and deoxyribo (dT) phosphoramidites, 2-[2-(4,4’-dimethoxytrityloxy)ethylsulfonyl]ethyl-(2-cyanoethyl)-(*N*,*N*-diisopropyl)-phosphoramidite (CPR, Chemical Phosphorylation Reagent) were purchased from Glen Research Inc. (Sterling, VA, USA). Propargylamine, 1,6-diaminohexane, (pyrene-1-yl-methyl)amine hydrochloride, *p*-anisic (4-methoxybenzoic) acid, *N*,*N*′-dicyclohexylcarbodiimide (DCC), 1-hydroxybenzotriazole hydrate (HOBt), *N*,*N*-diisopropylethylamine (DIPEA), α-GalNac-azide, and 1 M TBAF solution in THF were purchased from Sigma-Aldrich (St. Louis, MO, USA), *N*-Boc-1,6-diaminohexane hydrochloride and ethoxytrimethylsilane were obtained from Alfa Aesar (Heysham, UK); cholesterol chloroformate and oleylamine were obtained from Acros Organics (Geel, Belgium); 3-amino-1-propanol, triphenylphosphine (PPh_3_), and 2,2′-dipyridyl disulfide ((PyS)_2_) were obtained from Fluka (St. Louis, MO, USA). FAM-NHS, FAM-azide, 10 mM Cu(II)-TBTA Stock in 55% DMSO, and ascorbic acid were purchased from Lumiprobe (Moscow, Russia). All solvents (THF, DMSO, CH_3_CN (various vendors)) were dried by 3 Å molecular sieves or by distillation and stored over CaH_2_. Small molecule ligands were analyzed by thin-layer chromatography (TLC) using DC-Alufolien Kieselgel 60 F_254_ plates (Merck, Darmstadt, Germany) at 254 nm ultraviolet light.

### 3.2. Physical Measurements

AVANCE III 400 and 300 NMR spectrometers (Bruker Corporation, Billerica, MA, USA) were used to record the ^1^H-NMR spectra of the small molecule ligands, and CDCl_3_ was used as the solvent.

A MALDI-TOF Autoflex Speed mass spectrometer (Bruker Corporation, Billerica, MA, USA) or an Agilent G6410A LC-MS/MS Instrument (Agilent Technologies, Santa Clara, CA, USA) was used for the recording of mass spectra.

A NanoDrop 1000 spectrophotometer (Thermo Fisher Scientific, Waltham, MA, USA) was used to measure the oligonucleotide solutions’ optical densities.

After analytical gel-electrophoresis, the gels were either stained with a Stains-all dye for qualitative visualization or stained with ethidium bromide and quantified using the E-Box (Vilber, Marne-la-Vallée, France).

### 3.3. Preparation of Amino-Containing Compounds

Cholesteryl-6-aminohexylcarbamate (**I**) was prepared according to [40]. The yield of (**I**) was 70%, Rf 0.02 (TLC, 10% EtOH in CH_2_Cl_2_). ^1^H-NMR (300 MHz, CDCl_3_, ppm): 2.81 (m, 2H, NH_2_CH_2_-), 3.13 (t, 2H, -CONH-CH_2_-), 4.46 (m, 1H, oxycyclohexyl), 5.34 (s, 1H, alkenyl) (Supplementary Table S3).

*N*-(6-Aminohexyl)-4-methoxybenzamide (**II**)

4-Methoxybenzoic acid (0.3 g, 2.0 mmol) was dissolved in CH_2_Cl_2_ (12 mL) and simultaneously DCC (0.8 g, 4.0 mmol), previously dissolved in CH_2_Cl_2_ (10 mL), and HOBt (0.5 g, 4.0 mmol) was added, by analogy with [41]. The reaction was monitored by TLC (5% EtOH in CH_2_Cl_2_). After 16 h of shaking at room temperature, the reaction mixture was centrifuged and separated from the precipitate. The resulting derivative in solution was added to a solution of mono-*N*-Boc-protected hexamethylenediamine (1 g, 4.0 mmol) and abs. DIPEA (1 mL) in CH_2_Cl_2_ (5 mL). After 16 h of shaking at room temperature, the reaction mixture was evaporated, dissolved in 20 mL of CH_2_Cl_2_, and extracted with water (3 × 20 mL). Anhydrous Na_2_SO_4_ was used to dry the organic layer, which was then completely evaporated in vacuum to dryness. The substance was separated using column chromatography, then dried using evaporation. The yield was 41%, Rf 0.56. ^1^H-NMR (400 MHz, CDCl_3_, ppm): 1.27–1.49 (m, 15H, -CH_2_- and -C-(CH_3_)_3_), 1.57 (m, 2H, -CH_2_-), 3.09 (dd, 2H, -CH_2_-NHBoc), 3.39 (dd, 2H, -CONH-CH_2_-), 3.81 (s, 3H, -O-CH_3_), 6.89 (m, 2H, -CH-, benzene ring), 7.74 (d, 2H, -CH-, benzene ring) (Supplementary Table S3).

To remove the Boc-protecting group, mono-*N*-Boc-protected *N*-(6-aminohexyl)-4-methoxybenzamide was dissolved in 10 mL of CH_2_Cl_2_ and formic acid (1 mL) was added to the solution, according to [42]. After 2 h of shaking at room temperature, the reaction mixture was evaporated, dissolved in 30 mL of CH_2_Cl_2_, and washed with 0.1 M NaOH saturated with NaCl (4 × 20 mL). Anhydrous Na_2_SO_4_ was used to dry the organic layer, which was then completely evaporated in a vacuum until dry. The yield was 83%, Rf 0.02. ^1^H-NMR (400 MHz, CDCl_3_, ppm): 1.24–1.51 (m, 6H, -CH_2_-), 1.58 (m, 2H, -CH_2_-), 2.98 (m, 2H, NH_2_CH_2_-), 3.41 (m, 2H, -CONH-CH_2_-), 3.82 (s, 3H, -O-CH_3_), 6.90 (d, 2H, -CH-, benzene ring), 7.71 (d, 2H, -CH-, benzene ring) (Supplementary Table S3).

### 3.4. Synthesis of Polymer-Bound Oligonucleotides

Oligodeoxyribonucleotides, oligoribonucleotides, and oligo(2′-*O*-methylribonucleotides) were synthesized, as described in our previous work [40] (Supplementary Experimental Section S1). 5′-Phosphorylation of support-bound oligonucleotides was carried out as a standard automatic phosphoramidite cycle with the use of CPR phosphoramidite (0.1 M in anhydrous CH_3_CN); coupling time was 10 min. The subsequent solid-phase conjugation was carried out using polymer-bound DMTr-off oligonucleotides (see Section 3.5).

### 3.5. Solid-Phase Synthesis of Oligonucleotide Conjugates

BSA/DBU treatment to remove protecting groups from phosphates: removal of protecting groups from the internucleotide and 5′-terminal phosphates was carried out by analogy with [31]. The support-bound oligonucleotide was treated with 400 µL of THF/BSA (1/1, *v*/*v*) mixture for 30 min when shaken at room temperature, followed by the addition of 21 µL of DBU and shaking for next 30 min at room temperature. After that, the support was successively washed with THF (3 × 200 µL), CH_3_CN (3 × 200 µL), CH_2_Cl_2_ (3 × 200 µL) and air dried.

Synthesis of monoconjugates (**1–7, 9–12, 14–17**) (see Figure 1): Protecting groups were removed from the internucleotide and 5′-terminal phosphates of oligonucleotides, as described above. Then, Ph_3_P (7.9 mg, 0.03 mmol), (PyS)_2_ (6.6 mg, 0.03 mmol), DMAP (5.9 mg, 0.05 mmol), 200 μL abs. DMSO were added to the polymerized oligonucleotide (5–10 mg, 0.15–0.3 µmol) and left to shake for 20 min at 37 °C. The solution was decanted; the support was washed with abs. DMSO (3 × 250 µL). The solution of an amino-containing small molecules (cholesteryl-6-aminohexylcarbamate (**I**) (4.0 mg, 7.5 µmol) in 400 μL of CH_2_Cl_2_; 1-pyrenmethylamine hydrochloride (2.0 mg, 7.5 µmol) in 400 μL of DMSO/DIPEA mixture (4/1, *v*/*v*); *N*-(6-aminohexyl)-4-methoxybenzamide (**II**) (1.9 mg, 7.5 µmol) in 400 µL CH_2_Cl_2_; oleylamine (5.0 μL, 15 µmol) in 400 µL CH_2_Cl_2_; 1,6-diaminohexane (1.7 mg, 15 µmol) in 400 µL CH_2_Cl_2_; propargylamine (1.0 μL, 15 µmol) in 400 µL THF; 3-amino-1-propanol (1.1 μL, 15 µmol) in 400 μL of THF) was added to a 5′-phosphate activated oligonucleotide on a polymer carrier and left stirring for 16 h at 37 °C. At the end of the reaction, the solutions were decanted, and the polymer was washed with THF, CH_2_Cl_2_ or DMSO (3 × 300 µL), acetone (2 × 200 µL), and air dried. Next, unblocking, analysis of the reaction mixture by analytical gel electrophoresis and RP-HPLC and isolation by preparative gel electrophoresis were carried out (see Section 3.6).

### 3.6. Deprotection and Isolation of the Oligonucleotides and Their Conjugates

The oligonucleotide conjugates were cleaved from the support and deprotected by 40% aq.CH_3_NH_2_ (300 μL) for 2 h at room temperature, followed by Speedvac concentration. 2′-*O*-TBDMS groups were removed upon treatment with 1 M TBAF in THF (200 μL) overnight at room temperature, followed by the addition of 1 M TEAAc (pH 7.0) (600 μL), removed THF by Speedvac concentrator, and desalted with C18-cartridge or Amicon Ultra 3K (Millipore, Burlington, MA, USA). Unmodified control oligonucleotides were cleaved from the support and deprotected in the same way. 2′-*O*-TBDMS groups were removed upon treatment with a mixture of NMP/TEA·3HF/TEA (150/100/75, *v*/*v*/*v*) for 1.5 h at 65 °C, followed by treatment with ethoxytrimethylsilane. Deprotected oligonucleotides and their conjugates were isolated by 12% denaturing polyacrylamide gel electrophoresis (PAGE), followed by elution from the gel with 0.3 M NaClO_4_ solution, desalted with Amicon Ultra 3K, and precipitated as sodium salts. The total yields per the first nucleotide base are shown in Table 1. The purified oligonucleotide conjugates were characterized by RP-HPLC, PAGE and mass spectrometry (Table 1, Supplementary Table S2, Figures S1–S4).

### 3.7. Synthesis of Biotin Conjugate (**8**) Using NHS Esters

Biotin derivative (**8**) was obtained according to the NHS protocol of the reagent supplier (Lumiprobe, Moscow, Russia). The solution of the Biotin-NHS (0.5 mg, 1.1 mmol) in DMSO (80 μL) was added to the amino-modified oligonucleotide (**7**) (150 nmol) in 0.5 M Tris-HCl, pH 8.3 (20 μL). The mixture was incubated for 2 h at room temperature, precipitated with 2% solution of NaClO_4_ in acetone, washed with acetone, and air dried. The reaction mixture was dissolved and analyzed using PAGE and RP-HPLC (Supplementary Figure S2). Isolation was carried out as described above. The total yield per the first nucleotide base is shown in Table 1.

### 3.8. Synthesis of Conjugates (**13, 18**) Using Click-Chemistry

Triethylammonium acetate buffer (pH 7.0), 10 mM FAM-azide, or GalNac-azide in DMSO (20 μL), 5 mM ascorbic acid solution in water and 10 mM Cu(II)-TBTA stock in 55% DMSO were added to the water solution of 5′-alkyne-modified oligonucleotides (**12** or **17**) (100 nmol), according to the protocol of the click reagent supplier (Lumiprobe, Moscow, Russia). The reaction mixtures were incubated overnight at room temperature. The oligonucleotide conjugates were precipitated with 2% NaClO_4_ in acetone and washed with acetone. The pellet was air-dried, dissolved in water, and analyzed by RP-HPLC and/or PAGE (Supplementary Figure S3). The conversion of the oligonucleotide to the conjugate was almost quantitative, according to the PAGE and RP-HPLC (Supplementary Figure S3). Isolation was carried out, as described above. The total yields per the first nucleotide base are shown in Table 1.

### 3.9. RP-HPLC Analysis of the Oligonucleotide and Their Conjugates

Reversed-phase HPLC analysis of the oligonucleotides and their conjugates was performed on an Alphachrom A-02 high-performance liquid chromatograph (EcoNova, Novosibirsk, Russia) with the use of a ProntoSil-120-5-C18 AQ (75 × 2.0 mm, 5.0 μm) column, applying a gradient elution from 0% to 50% (25 min) of CH_3_CN in 0.02 M triethylammonium acetate buffer, pH 7.0, at a flow rate 100 μL per min.

### 3.10. Stability of the P-N-Bond within the Oligonucleotide Conjugates (**14–16, 18**) at Different pH Values

Conjugates of 5′-p-siDmS oligonucleotide (**14–16**, **18**) with *N*-(6-aminohexyl)-4-methoxybenzamide, cholesterol-6-aminohexylcarbamate, oleylamine and GalNAc (27 nmol) were kept in a NaOAc-buffer (0.05M, 90 μL) with pH values 6.0, 5.2, and 4.5 at 37 °C. After 1, 2, 4, 6, and 24 h, 15 μL aliquots were taken, and the oligonucleotides were precipitated with 2% NaClO_4_ in acetone and washed with acetone. The pellet was air-dried, dissolved in water, analyzed by denatured gel electrophoresis, and stained with ethidium bromide. The resulting electropherograms were digitized and processed using the Quantity One program (BioRad, Hercules, CA, USA). Each experiment was repeated at least three times. The statistical analyses were performed using GraphPad Prism 6.01 (GraphPad Software, San Diego, CA, USA). The outcome variables are expressed as means ± standard deviations (SDs).

## 4. Conclusions

We proposed a new, convenient solid-phase approach for attaching various transporting small molecules to the 5′-end of an oligonucleotide via the biodegradable, acid-labile phosphoramide bond. The method is simple and efficient and allows for the fine-tuning of the ratio of different solvents for a desired ligand over a wide range. Moreover, the unreacted reaction components can easily be removed at any step by washing since the conjugate is attached to the support during the whole synthesis. The method is based on the activation of the 5′-terminal phosphate of a protected support-bound oligonucleotide, followed by interaction with a small molecule bearing a primary amino group. We demonstrated the advantages of this approach in the synthesis of a series of oligonucleotide 5′-conjugates with different ligands, such as cholesterol, aliphatic amine, *N*-acetylgalactosamine (GalNAc), and *p*-anisic acid (anisamide). The obtained conjugates were characterized by HPLC, analytical PAGE, and mass-spectrometry. The effective release of the oligonucleotide from the small molecules was shown under mildly acidic conditions that are close to the pH value within endosomes/lysosomes. Our subsequent studies will include a series of in vitro experiments to examine the influence of the small molecules themselves and also the type of the linker (stable/labile) on the biological activity of functional nucleic acids bearing transport ligands. The developed method is compatible with various types of oligonucleotides (deoxyribo-, 2′-O-methylribo-, ribo- and others) and can be further used for obtaining the conjugates of antisense oligonucleotides, siRNAs, miRNAs, or aptamers with transporting ligands to improve their cell delivery and cargo release inside the cell.

## Figures and Tables

**Table 1:** Synthesized conjugates of oligonucleotides and their characteristics.

№	Oligonucleotide Conjugate, 5′-3′	RP HPLC Retention Time, min ^1^	Molecular Weight		Yield, % ^3^
			Calculated	Experimental ^2^	
1	*MB*-**L_6_**-NH-p-d(TTTTTTT)	12.88 (+3.64)	2379.7	2378.5	19
2	*Chol*-C(O)-**L_6_**-NH-p-d(TTTTTTT)	24.05 (+14.81)	2658.2	2656.5	18
3	*Oleyl*-NH-p-d(TTTTTTT)	23.53 (+14.29)	2396.8	2395.5	24
4	CH≡C-CH_2_-NH-p-d(TTTTTTT)	9.93 (+0.69)	2184.4	2183.0	23
5	*Pyr*-CH_2_-NH-p-d(TTTTTTT)	15.21 (+5.97)	2360.3	2358.9	23
6	HO-(CH_2_)_3_-NH-p-d(TTTTTTT)	9.63 (+0.39)	2204.4	2203.2	22
7	NH_2_-(CH_2_)_6_-NH-p-d(TTTTTTT)	9.43 (+0.19)	2245.6	2244.0	18
8	*Biot*-NH-(CH_2_)_6_-NH-p-d(TTTTTTT)	11.88 (2.64)	2471.6	2470.2	17 ^5,^*
9	CH_3_-NH-p-d(TTTTTTT)	9.55 (+0.31)	2161.1	2159.7	21 *
10	*Oleyl*-NH-p-G^m^G^m^C^m^U^m^U^m^G^m^A^m^C^m^A^m^	17.12 (+7.01)	3310.3	3309.0	18
11	*MB*-**L_6_**-NH-p-G^m^G^m^C^m^U^m^U^m^G^m^A^m^C^m^A^m^	12.01 (+1.9)	3293.3	3291.3	18
12	CH≡C-CH_2_-NH-p-G^m^G^m^C^m^U^m^U^m^G^m^A^m^C^m^A^m^	10.39 (+0.28)	3098.1	3096.2	21
13	*FAM-click*-CH_2_-NH-p-G^m^G^m^C^m^U^m^U^m^G^m^A^m^C^m^A^m^	11.81 (+1.70)	3555.5	3554.5	21 ^5,^*
14	*MB*-**L_6_**-NH-p-GGCUUGACAAGUUGUAUAUGG^m^	n/a ^4^	7080.4	7080.28	20
15	*Chol*-C(O)-**L_6_**-NH-p-GGCUUGACAAGUUGUAUAUGG^m^	n/a ^4^	7358.9	7358.98	17
16	*Oleyl*-NH-p-GGCUUGACAAGUUGUAUAUGG^m^	n/a ^4^	7097.6	7097.2	21
17	CH≡C-CH_2_-NH-p-GGCUUGACAAGUUGUAUAUGG^m^	n/a ^4^	6885.1	6886.4	20
18	*GalNAc-click*-CH_2_-NH-p-GGCUUGACAAGUUGUAUAUGG^m^	n/a ^4^	7263.7	7263.87	19 ^5,^*

^1^ For the RP-HPLC conditions, see Section 3: Materials and Methods. The difference between the retention times for the nonmodified controls 5′-p-d(TTTTTTT) (9.24 min) or 5′-p-G^m^G^m^C^m^U^m^U^m^G^m^A^m^C^m^A^m^ (10.11 min) are given in the brackets. ^2^ Obtained by ESI or MALDI-TOF mass spectrometry. ^3^ The yields of conjugates after deblocking and isolation were calculated based on the molar amount of the first support-bound nucleoside. ^4^ Not available, characterized by PAGE only. ^5^ After all conjugations and isolation. * For the description, see Supplementary material. *Chol*-C(O)-**L_6_**-NH–, cholesteryl-6-aminohexylcarbamate residue; *Oleyl*-NH–, oleylamine residue; *Pyr*-CH_2_-NH–, pyrenemethylamine residue; *MB*-**L_6_**-NH-p-, *N*-(6-aminohexyl)-4-methoxybenzamide residue; NH_2_-(CH_2_)_6_-NH-, 1,6-diaminohexane residue; HO-(CH_2_)_3_-NH-, 3-amino-1-propanole residue; CH≡C-CH_2_-NH–, propargylamine residue; *Biot*-, Biotin residue (Supplementary Figure S2); *FAM-click*-CH_2_-NH-, FAM residue with 1,2,3-triazole linker (Supplementary Figure S3); *GalNAc-click*, GalNAc residue with 1,2,3-triazole linker (Supplementary Figure S3); -p-, -P(O)(OH)-; **L_6_**-, -NH(CH_2_)_6_-; N, ribonucleotide; N^m^, 2′-O-methylribonucleotide; d(N), deoxyribonucleotide.

## Data Availability

Not applicable.

## Supplementary Materials

The following supporting information can be downloaded at: https://www.mdpi.com/article/10.3390/molecules28041904/s1, Table S1: The amino ligands used for solid-phase attachment to oligonucleotides and selected optimal solvents for this reaction; Figure S1: Electrophoretic analysis of reaction mixtures upon solid-phase conjugation; Figure S2: Functionalization of the 5′-amino-modified oligonucleotide (**7**) with Biotin N-hydroxysuccinimide ester; Figure S3: Attachment of FAM or α-GalNAc azides to the 5′-alkyne-modified oligonucleotide (**12**) or (**17**) using “click”-chemistry reaction; Table S2: Representative ESI or MALDI-TOF mass spectra of the 5′-conjugates of oligonucleotides; Table S3: ^1^H-NMR spectra of amino-containing ligands; Figure S4: Full-size images of electropherograms after PAGE analysis and Stains-all staining for 5′-phosphorylated oligonucleotides and their conjugates (**1–18**); Experimental Section S1: Automated synthesis of polymer-bound oligonucleotides; Table S4: Stability of the P-N-bond within the oligonucleotide conjugates (**14–16, 18**) at different pH values.

## Dedication

Dedicated to the memory of our colleague, Alya G. Venyaminova (ICBFM, Novosibirsk, Russia), an expert in RNA chemistry and applications.

## Sample Availability

Samples of the compounds are not available from the authors.

## References

[1] Laganà A., Shasha D., Croce C.M. (2014). Synthetic RNAs for Gene Regulation: Design Principles and Computational Tools. Front. Bioeng. Biotechnol..

[2] Sridharan K., Gogtay N.J. (2016). Therapeutic Nucleic Acids: Current Clinical Status. Br. J. Clin. Pharmacol..

[3] Panigaj M., Johnson M.B., Ke W., McMillan J., Goncharova E.A., Chandler M., Afonin K.A. (2019). Aptamers as Modular Components of Therapeutic Nucleic Acid Nanotechnology. ACS Nano.

[4] Smith C.I.E., Zain R. (2019). Therapeutic Oligonucleotides: State of the Art. Annu. Rev. Pharmacol. Toxicol..

[5] Jani S., Ramirez M.S., Tolmasky M.E. (2021). Silencing Antibiotic Resistance with Antisense Oligonucleotides. Biomedicines.

[6] Bajan S., Hutvagner G. (2020). RNA-Based Therapeutics: From Antisense Oligonucleotides to MiRNAs. Cells.

[7] Winkler J. (2013). Oligonucleotide Conjugates for Therapeutic Applications. Ther. Deliv..

[8] Juliano R.L. (2016). The Delivery of Therapeutic Oligonucleotides. Nucleic Acids Res..

[9] Nakagawa O., Ming X., Huang L., Juliano R.L. (2010). Targeted Intracellular Delivery of Antisense Oligonucleotides via Conjugation with Small-Molecule Ligands. J. Am. Chem. Soc..

[10] Springer A.D., Dowdy S.F. (2018). GalNAc-SiRNA Conjugates: Leading the Way for Delivery of RNAi Therapeutics. Nucleic Acid Ther..

[11] Dong Y., Siegwart D.J., Anderson D.G. (2019). Strategies, Design, and Chemistry in SiRNA Delivery Systems. Adv. Drug Deliv. Rev..

[12] Benizri S., Gissot A., Martin A., Vialet B., Grinstaff M.W., Barthélémy P. (2019). Bioconjugated Oligonucleotides: Recent Developments and Therapeutic Applications. Bioconjug. Chem..

[13] Hawner M., Ducho C. (2020). Cellular Targeting of Oligonucleotides by Conjugation with Small Molecules. Molecules.

[14] Zhang Y., Sun C., Wang C., Jankovic K.E., Dong Y. (2021). Lipids and Lipid Derivatives for RNA Delivery. Chem. Rev..

[15] Wolff J.A., Rozema D.B. (2008). Breaking the Bonds: Non-Viral Vectors Become Chemically Dynamic. Mol. Ther..

[16] Leriche G., Chisholm L., Wagner A. (2012). Cleavable Linkers in Chemical Biology. Bioorg. Med. Chem..

[17] Choy C.J., Ley C.R., Davis A.L., Backer B.S., Geruntho J.J., Clowers B.H., Berkman C.E. (2016). Second-Generation Tunable PH-Sensitive Phosphoramidate-Based Linkers for Controlled Release. Bioconjug. Chem..

[18] Le Corre S.S., Berchel M., Couthon-Gourvès H., Haelters J.-P., Jaffrès P.-A. (2014). Atherton–Todd Reaction: Mechanism, Scope and Applications. Beilstein J. Org. Chem..

[19] Vlaho D., Fakhoury J.F., Damha M.J. (2018). Structural Studies and Gene Silencing Activity of SiRNAs Containing Cationic Phosphoramidate Linkages. Nucleic Acid Ther..

[20] Cooke L.A., Frauendorf C., Gîlea M.A., Holmes S.C., Vyle J.S. (2006). Solid-Phase Synthesis of Terminal Oligonucleotide–Phosphoramidate Conjugates. Tetrahedron Lett..

[21] Gołębiewska J., Rachwalak M., Jakubowski T., Romanowska J., Stawinski J. (2018). Reaction of Boranephosphonate Diesters with Amines in the Presence of Iodine: The Case for the Intermediacy of H-Phosphonate Derivatives. J. Org. Chem..

[22] Kupryushkin M.S., Apukhtina V.S., Vasilyeva S.V., Pyshnyi D.V., Stetsenko D.A. (2015). A New Simple and Convenient Method for Preparation of Oligonucleotides Containing a Pyrene or a Cholesterol Moiety. Russ. Chem. Bull..

[23] Derzhalova A., Markov O., Fokina A., Shiohama Y., Zatsepin T., Fujii M., Zenkova M., Stetsenko D. (2021). Novel Lipid-Oligonucleotide Conjugates Containing Long-Chain Sulfonyl Phosphoramidate Groups: Synthesis and Biological Properties. Appl. Sci..

[24] Dovydenko I.S., Kupryushkin M.S., Pyshnyi D.V., Apartsin E.K. (2018). A Convenient Solid Phase Approach to Obtain Lipophilic 5′-Phosphoramidate Derivatives of DNA and RNA Oligonucleotides. Nucleosides Nucleotides Nucleic Acids.

[25] Jeong J.H., Kim S.W., Park T.G. (2003). Novel Intracellular Delivery System of Antisense Oligonucleotide by Self-Assembled Hybrid Micelles Composed of DNA/PEG Conjugate and Cationic Fusogenic Peptide. Bioconjug. Chem..

[26] Wang T.-P., Ko N.C., Su Y.-C., Wang E.-C., Severance S., Hwang C.-C., Shih Y.T., Wu M.H., Chen Y.-H. (2012). Advanced Aqueous-Phase Phosphoramidation Reactions for Effectively Synthesizing Peptide–Oligonucleotide Conjugates Trafficked into a Human Cell Line. Bioconjug. Chem..

[27] Mukaiyama T., Hashimoto M. (1972). Synthesis of Oligothymidylates and Nucleoside Cyclic Phosphates by Oxidation-Reduction Condensation. J. Am. Chem. Soc..

[28] Zarytova V., Ivanova E., Venyaminova A. (1998). Design of Functional Diversity in Oligonucleotides via Zwitter-Ionic Derivatives of Deprotected Oligonucleotides. Nucleosides Nucleotides.

[29] Grimm G.N., Boutorine A.S., Helene C. (2000). Rapid Routes of Synthesis of Oligonucleotide Conjugates from Non-Protected Oligonucleotides and Ligands Possessing Different Nucleophilic or Electrophilic Functional Groups. Nucleosides Nucleotides Nucleic Acids.

[30] Novopashina D.S., Totskaya O.S., Kholodar’ S.A., Meshchaninova M.I., Ven’yaminova A.G. (2008). Oligo(2′-O-Methylribonucleotides) and Their Derivatives: III. 5′-Mono- and 5′-Bispyrenyl Derivatives of Oligo(2′-O- Methylribonucleotides) and Their 3′-Modified Analogues: Synthesis and Properties. Russ. J. Bioorganic Chem..

[31] Krasheninina O.A., Novopashina D.S., Lomzov A.A., Venyaminova A.G. (2014). 2′-Bispyrene-Modified 2′-O-Methyl RNA Probes as Useful Tools for the Detection of RNA: Synthesis, Fluorescent Properties, and Duplex Stability. ChemBioChem.

[32] Lönnberg H. (2009). Solid-Phase Synthesis of Oligonucleotide Conjugates Useful for Delivery and Targeting of Potential Nucleic Acid Therapeutics. Bioconjug. Chem..

[33] Cedillo I., Chreng D., Engle E., Chen L., McPherson A., Rodriguez A. (2017). Synthesis of 5′-GalNAc-Conjugated Oligonucleotides: A Comparison of Solid and Solution-Phase Conjugation Strategies. Molecules.

[34] Singh Y., Murat P., Defrancq E. (2010). Recent Developments in Oligonucleotide Conjugation. Chem. Soc. Rev..

[35] Raouane M., Desmaële D., Urbinati G., Massaad-Massade L., Couvreur P. (2012). Lipid Conjugated Oligonucleotides: A Useful Strategy for Delivery. Bioconjug. Chem..

[36] Gooding M., Malhotra M., Evans J.C., Darcy R., O’Driscoll C.M. (2016). Oligonucleotide Conjugates—Candidates for Gene Silencing Therapeutics. Eur. J. Pharm. Biopharm..

[37] Dasargyri A., Kümin C.D., Leroux J.-C. (2017). Targeting Nanocarriers with Anisamide: Fact or Artifact?. Adv. Mater..

[38] Hayashi T., Su T. (2004). Sigma-1 Receptors at Galactosylceramide-Enriched Lipid Microdomains Regulate Oligodendrocyte Differentiation. Proc. Natl. Acad. Sci. USA.

[39] Qu D., Jiao M., Lin H., Tian C., Qu G., Xue J., Xue L., Ju C., Zhang C. (2020). Anisamide-Functionalized PH-Responsive Amphiphilic Chitosan-Based Paclitaxel Micelles for Sigma-1 Receptor Targeted Prostate Cancer Treatment. Carbohydr. Polym..

[40] Meschaninova M.I., Novopashina D.S., Semikolenova O.A., Silnikov V.N., Venyaminova A.G. (2019). Novel Convenient Approach to the Solid-Phase Synthesis of Oligonucleotide Conjugates. Molecules.

[41] Jain P.K., Friedman S.H. (2018). The ULTIMATE Reagent: A Universal Photocleavable and Clickable Reagent for the Regiospecific and Reversible End Labeling of Any Nucleic Acid. ChemBioChem.

[42] Coffey D.S., McDonald A.I., Overman L.E., Rabinowitz M.H., Renhowe P.A. (2000). A Practical Entry to the Crambescidin Family of Guanidine Alkaloids. Enantioselective Total Syntheses of Ptilomycalin A, Crambescidin 657 and Its Methyl Ester (Neofolitispates 2), and Crambescidin 800. J. Am. Chem. Soc..

[43] Sekine M., Tsuruoka H., Iimura S., Kusuoku H., Wada T., Furusawa K. (1996). Studies on Steric and Electronic Control of 2‘–3‘ Phosphoryl Migration in 2‘-Phosphorylated Uridine Derivatives and Its Application to the Synthesis of 2‘-Phosphorylated Oligouridylates. J. Org. Chem..

[44] Petrova N.S., Chernikov I.V., Meschaninova M.I., Dovydenko I.S., Venyaminova A.G., Zenkova M.A., Vlassov V.V., Chernolovskaya E.L. (2012). Carrier-Free Cellular Uptake and the Gene-Silencing Activity of the Lipophilic SiRNAs Is Strongly Affected by the Length of the Linker between SiRNA and Lipophilic Group. Nucleic Acids Res..

[45] Bramsen J.B., Laursen M.B., Damgaard C.K., Lena S.W., Babu B.R., Wengel J., Kjems J. (2007). Improved Silencing Properties Using Small Internally Segmented Interfering RNAs. Nucleic Acids Res..

[46] Meschaninova M.I., Entelis N.S., Chernolovskaya E.L., Venyaminova A.G. (2021). A Versatile Solid-Phase Approach to the Synthesis of Oligonucleotide Conjugates with Biodegradable Hydrazone Linker. Molecules.

[47] Gilleron J., Querbes W., Zeigerer A., Borodovsky A., Marsico G., Schubert U., Manygoats K., Seifert S., Andree C., Stöter M. (2013). Image-Based Analysis of Lipid Nanoparticle–Mediated SiRNA Delivery, Intracellular Trafficking and Endosomal Escape. Nat. Biotechnol..

