# Supplementary material for: Convenient Solid-Phase Attachment of Small-Molecule Ligands to Oligonucleotides via a Biodegradable Acid-Labile P-N-Bond
Source: Molecules. 2023 Feb 16;28(4):1904. doi: 10.3390/molecules28041904 (PMC9961013; doi:10.3390/molecules28041904)
Supplement: Supplementary file 1 [file molecules-28-01904-s001.zip › molecules-2210994-supplementary.pdf]

molecules MDPI

Article

Convenient Solid-Phase Attachment of Small-Molecule Ligands to Oligonucleotides via a Biodegradable Acid-Labile P-N-Bond

Nadezhda O. Kropacheva [1,2], Arseniy A. Golyshkin [2], Mariya A. Vorobyeva [1] and Mariya I. Meschaninova [1,*]

[1] Institute of Chemical Biology and Fundamental Medicine, Siberian Branch, Russian Academy of Sciences, Novosibirsk 630090, Russia; n.kropacheva1@g.nsu.ru (N.O.K.); kuzn@niboch.nsc.ru (M.A.V.)
[2] Department of Natural Sciences, Novosibirsk State University, Novosibirsk 630090, Russia; a.golyshkin@g.nsu.ru
* Correspondence: mesch@niboch.nsc.ru; Tel.: +7-383-363-5129

Content

	Page
**Table S1**. The amino ligands used for solid-phase attachment to oligonucleotides and selected optimal solvents for this reaction	S2
**Figure S1**. Electrophoretic analysis of reaction mixtures upon solid-phase conjugation	S3
**Figure S2**. Functionalization of the 5'-amino modified oligonucleotide (**7**) with Biotin *N*-hydroxysuccinimide ester	S4
**Figure S3**. Attachment of FAM or α-GalNAc azides to the 5'-alkyne-modified oligonucleotide (**12**) or (**17**) using “click”-chemistry reaction	S5
**Table S2**. Representative ESI or MALDI-TOF mass spectra of the 5'-conjugates of oligonucleotides	S6
**Table S3**. ^{1}H-NMR spectra of amino containing ligands	S14
**Figure S4**. Full-size images of electropherograms after PAGE analysis and Stains-all staining for 5'-phosphorylated oligonucleotides and their conjugates (**1-18**)	S16
**Experimental Section S1.** Automated synthesis of polymer-bound oligonucleotides	S17
**Table S4**. Stability of the P-N-bond within the oligonucleotide conjugates (**14-16, 18**) at different pH values	S18

Table S1. The amino ligands used for solid-phase attachment to oligonucleotides and selected optimal solvents for this reaction.

Structure	Solvent	Molecular weight	References
**Pyrenemethylamine**<br>**(***Pyr*-CH_2-NH_2**)**<br>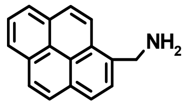	DMSO	231.29	Sigma-Aldrich
**1,6-Diaminohexane**<br>NH_2-$(CH_2)_6$-NH_2	CH_2Cl_2	116.21	Sigma-Aldrich
**3-Amino-1-propanol**<br>HO-$(CH_2)_3$-NH_2	THF	75.11	Sigma-Aldrich
**Propargylamine**<br>HC≡C-CH_2-NH_2	THF	55.08	Sigma-Aldrich
**Oleylamine**<br>**(***Oleyl*-NH_2**)**<br>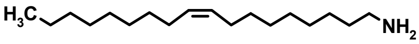	CH_2Cl_2	267.49	Sigma-Aldrich
**Cholesteryl-6-aminohexylcarbamate (I)**<br>(*Chol*-C(O)-**L**$_6$-NH_2, where **L**$_6$: -NH$(CH_2)_6$-)<br>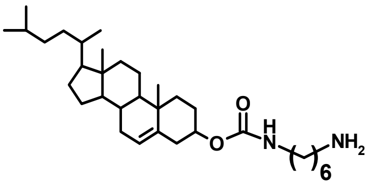	CH_2Cl_2	528.85	See Materials and Methods, Section 4.4
***N*-(6-Aminohexyl)-4-methoxybenzamide (II)**<br>(*MB*-**L**$_6$-NH_2, where **L**$_6$: -NH$(CH_2)_6$-)<br>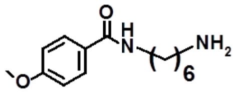	CH_2Cl_2	250.36	See Materials and Methods, Section 4.4

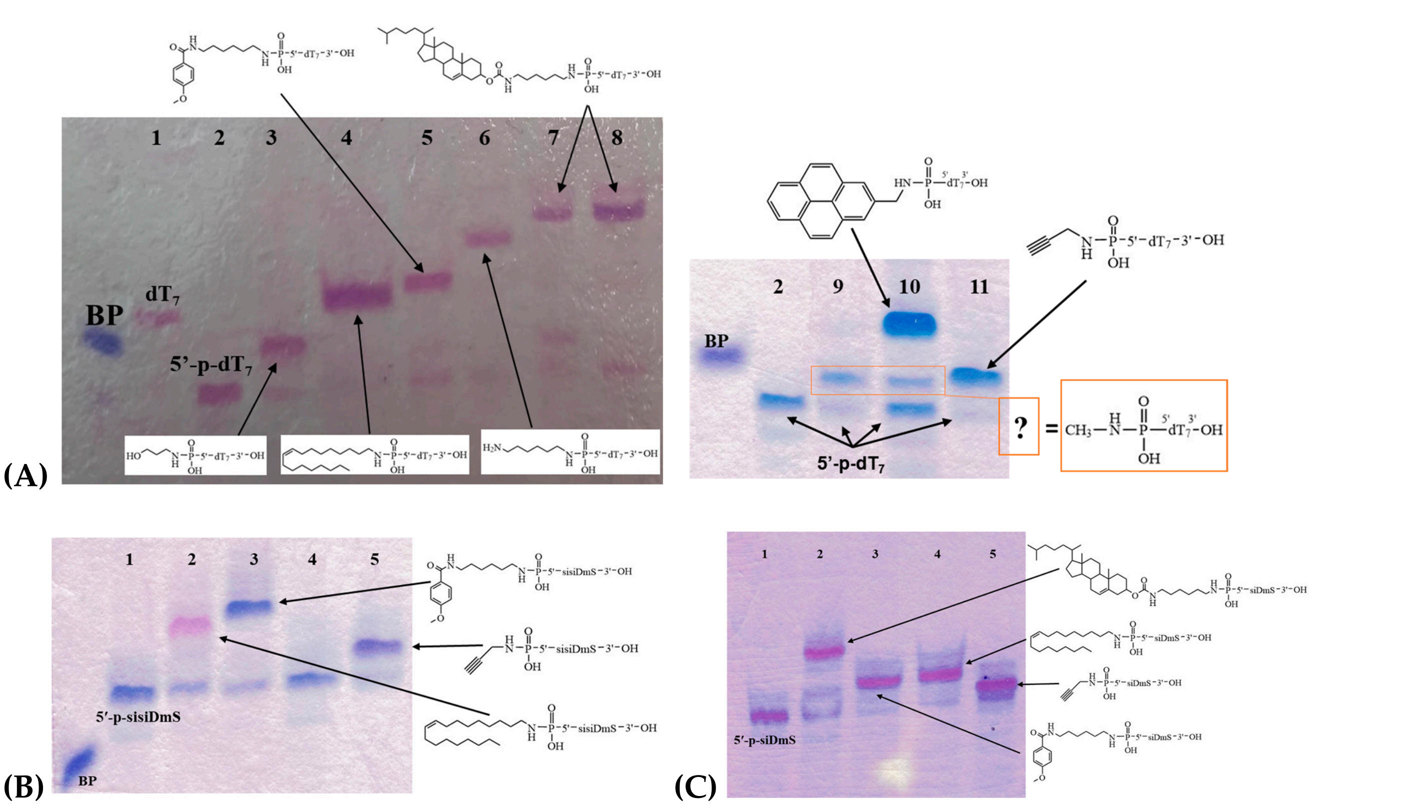

Figure S1. Electrophoretic analysis of reaction mixtures upon solid-phase conjugation.Analysis of reaction mixtures after solid-phase synthesis by PAGE: **(A)** line **1** - dT_7, line **2** - initial 5'-p-dT_7, line **3** - HO-$(CH_2)_3$-NH-p-dT_7 **(6)**, line **4** - *Oleyl*-NH-p-dT_7 **(3)**, line **5** - *MB*-**L_6**-NH-p-dT_7 **(1)**, line **6** - NH_2-$(CH_2)_6$-NH-p-dT_7 **(7)**, line **7** and **8** - *Chol*-C(O)-**L_6**-NH-p-dT_7 **(2)**, line **9** - CH_3-NH-p-dT_7 **(9)**, line **10** - *Pyr*-CH_2-NH-p-dT_7 **(5)**; line **11** - CH≡C-CH_2-NH-p-dT_7 **(4)**;**(B)** line **1** and **4** - initial 5'-p-sisiDmS, line **2 -** *Oleyl*-NH-p-sisiDmS **(11)**, line **3** - *MB*-**L_6**-NH-p-sisiDmS ***(i)***, line **5** - CH≡C-CH_2-NH-p-sisiDmS ***(i)***; **(C)** line **1** - initial 5'-p-siDmS, line **2** - *Chol*-C(O)-**L_6**-NH-p-siDmS **(15)**, line **3** - *MB*-**L_6**-NH-p-siDmS ***(i)***, line **4** - *Oleyl*-NH-p-siDmS **(16)**, line **5** - CH≡C-CH_2-NH-p-siDmS **(17)**. Conditions: 15% denaturating PAAG (7M urea, acrylamide/*N,N′*-methylene bis-acrylamide (19/1)) in TBE buffer. Gel stained with "Stains-all". BP – bromophenol blue.*Chol*-C(O)-**L_6**-NH-, cholesteryl-6-aminohexylcarbamate residue; *Oleyl*-NH-, oleylamine residue; *Pyr*-CH_2-NH-, pyrenemethylamine residue; *MB*-**L_6**-NH-, *N*-(6-aminohexyl)-4-methoxybenzamide residue; NH_2-$(CH_2)_6$-NH-, 1,6-diaminohexane residue; HO-$(CH_2)_3$-NH-, 3-amino-1-propanole residue; CH≡C-CH_2-NH-, propargylamine residue; -p-, -P(O)(OH)-; **L_6** -, -NH$(CH_2)_6$-; dT_7 = 5'-d(TTTTTTT); 5′-p-dT_7 = 5′-p-d(TTTTTTT); 5′-p-sisiDmS = 5′-p-$G^mG^mC^mU^mU^mG^mA^mC^mA^m$; 5′-p-siDmS = 5′-p-GGCUUGACAAGUUGUAUAUGG^m (d(N), deoxyribonucleotide; N^m, 2′-O-methylribonucleotide; N, ribonucleotide). Full-size images of electropherograms after analyses of reaction mixtures are given in Figure S4. .

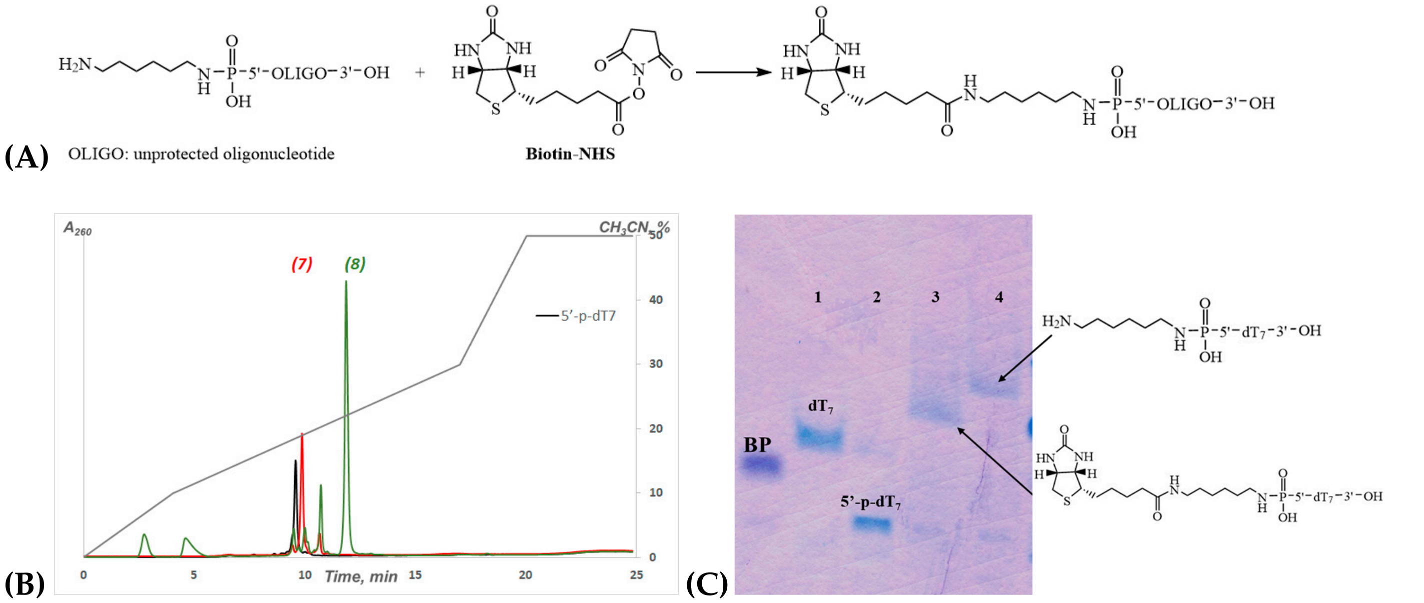

Figure S2. Functionalization of the 5'-amino modified oligonucleotide (7) with Biotin N-hydroxysuccinimide ester**. (A)** Scheme of the synthesis of Biotin-conjugate of 5′-p-dT7 (**8**). **(B)** RP HPLC analysis of reaction mixtures of initial oligonucleotide 5′-p-dT7 and conjugates (**7**) and (**8**). The degree of conversion of (*7*) to (*8*) was 82%. Conditions: Alphachrom A-02 high performance liquid chromatograph (EcoNova, Novosibirsk, Russia), ProntoSil-120-5-C18 AQ (75×2.0 mm, 5.0 μm) column, gradient elution from 0 to 50% (25 min) of acetonitrile in 0.02 M triethylammonium acetate buffer, pH 7.0, flow rate 100 μL per min, detection at 260 nm.**(C)** Analysis of reaction mixtures of 5′-p-dT7 and conjugates (**7**) and (**8**) by PAGE: line **1 -** deblocked reaction mixtures of dT7 oligonucleotide; line **2 -** deblocked reaction mixtures of 5′-p-dT7 oligonucleotide; line **3 -** reaction mixture after Biotin-NHS attachment to derivative (**7**) in solution to obtain conjugate (**8**); line **4** – deblocked reaction mixture after solid-phase attachment of 1,6-diaminohexane to the activated 5′-p-dT7 to obtain derivative (**7**). Conditions: 15% denaturating PAAG (7M urea, acrylamide/*N,N′*-methylene bis-acrylamide (19/1)) in TBE buffer. Gel stained with "Stains-all". BP – bromophenol blue. 5′-p-dT7 = 5′-p-d(TTTTTTT); -p-, -P(O)(OH)-; d(N), deoxyribonucleotide.Full-size images of electropherograms after analyses of reaction mixtures are given in Figure S4.

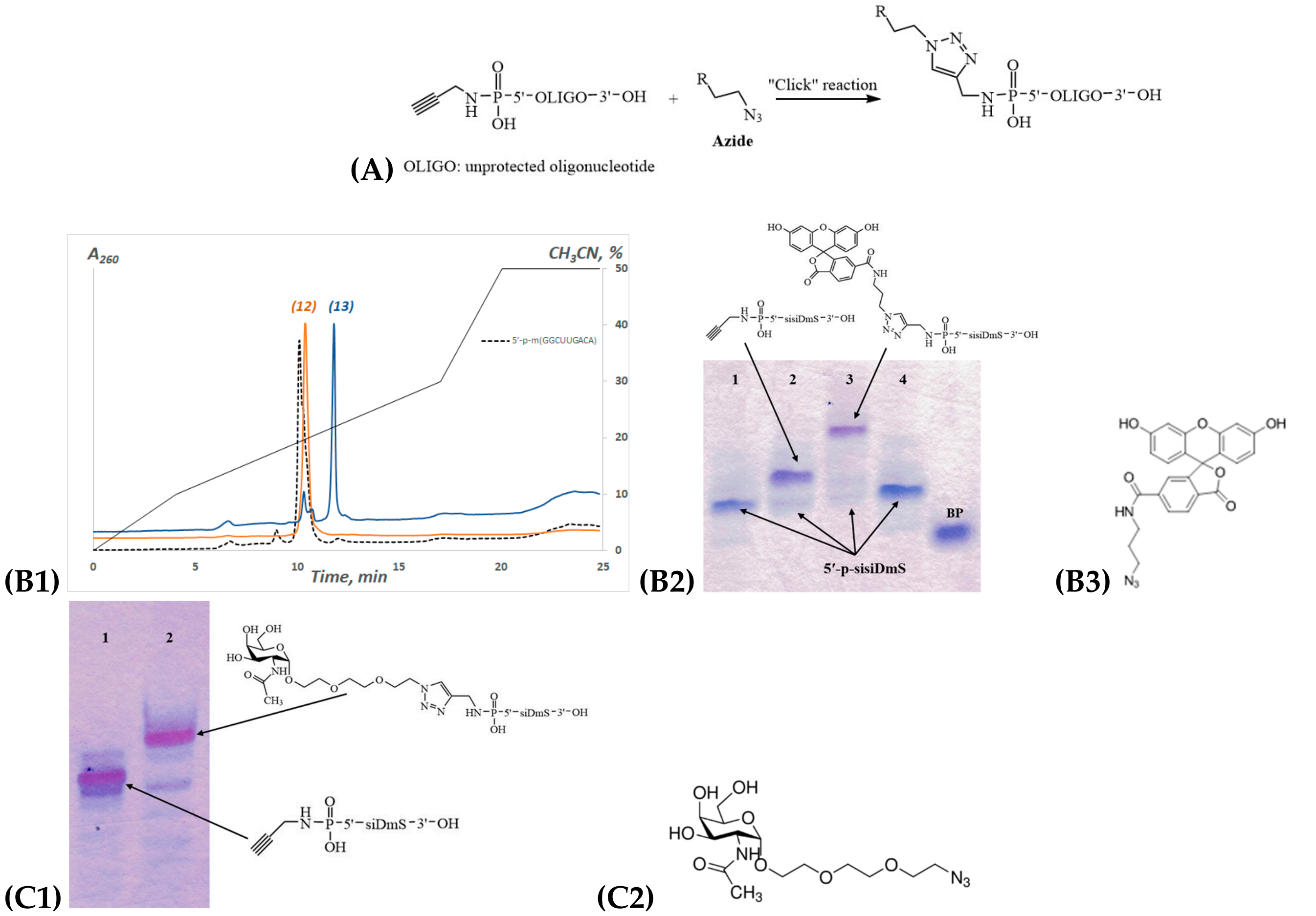

Figure S3. Attachment of FAM or α-GalNAc azides to the 5'-alkyne-modified oligonucleotide (12) or (17) using "click"-chemistry reaction.**(A)** Scheme of the synthesis of conjugates (**13**) and (**18**) using "click"-reaction of with 5'-propargylamine-modified oligonucleotides (*12*) and (*17*), respectively. **(B1)** Reverse phase-HPLC (RP-HPLC) analysis of reaction mixtures of the 5′-p-sisiDmS and conjugates (**12**) and (**13**). The degree of conversion of *(***12***)* to *(***13***)* was 87%. Conditions: Alphachrom A-02 high performance liquid chromatograph (EcoNova, Novosibirsk, Russia), ProntoSil-120-5-C18 AQ (75×2.0 mm, 5.0 μm) column, gradient elution from 0 to 50% (25 min) of acetonitrile in 0.02 M triethylammonium acetate buffer, pH 7.0, flow rate 100 μL per min, detection at 260 nm. **(B2)** Analysis of reaction mixtures of 5′-p-sisiDmS and conjugates (**12**) and (**13**) by PAGE: line **1** and **4 -** deblocked reaction mixtures of initial 5′-p-sisiDmS oligonucleotide; line **2** – deblocked reaction mixture after solid-phase attachment of propargylamine to the activated 5′-p-sisiDmS to obtain derivative (**12**); line **3 -** reaction mixture after FAM azide attachment to derivative (**12**) *via* "click"-chemistry in solution to obtain conjugate (**13**). **(B3)** Structure of FAM azide. **(C1)** Analysis of reaction mixtures of 5′-p-siDmS and conjugates (**17**) and (**18**) by PAGE: line **1 -** deblocked reaction mixtures of initial 5′-p-siDmS oligonucleotide; line **2** – deblocked reaction mixture after solid-phase attachment of propargylamine to the activated 5′-p-siDmS to obtain derivative (**17**); line **3 -** reaction mixture after α-GalNAc-PEG3 azide attachment to derivative (**17**) *via* "click"-chemistry in solution to obtain conjugate (**18**). **(C2)** Structure of α-GalNAc-PEG3-azide.Conditions of PAGE: 15% denaturating PAAG (7M urea, acrylamide/*N,N′*-methylene bis-acrylamide (19/1)) in TBE buffer. Gel stained with "Stains-all". BP – bromophenol blue. 5′-p-sisiDmS = 5'-p-$G^mG^mC^mU^mU^mG^mA^mC^mA^m$; 5′-p-siDmS = 5′-p-GGCUUGACAAGUUGUAUAUGG^m; -p-, -P(O)(OH)-; N, ribonucleotide; N^m, 2′-O-methylribonucleotide.Full-size images of electropherograms after analyses of reaction mixtures are given in Figure S4.

Table S2. Representative ESI or MALDI-TOF mass spectra of the 5'-conjugates of oligonucleotides.

(1) *MB*-**L$_6$**-NH-p-d(TTTTTTT)

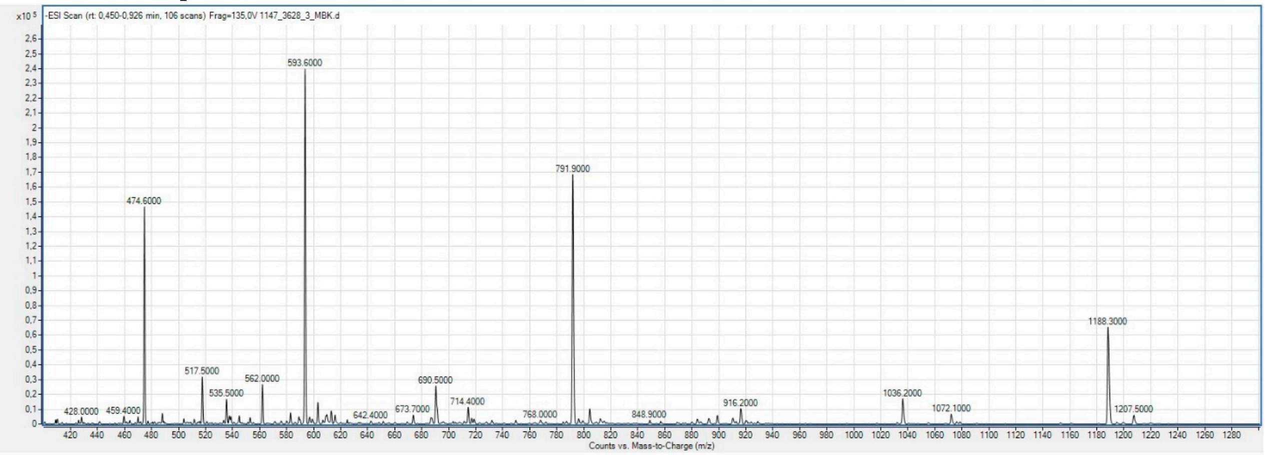

[H5] / 474.6	2378.0 →	M 2378.0
[H4] / 593.6	2378.4 →	M 2378.4
[H3] / 791.9	2378.8 →	M 2378.8
[H2] / 1188.3	2378.6 →	M 2378.6
		M$_{found}$ 2378.5

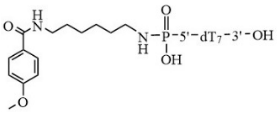

M$_{calcd}$ 2379.7

(2) *Chol*-C(O)-**L$_6$**-NH-p-d(TTTTTTT)

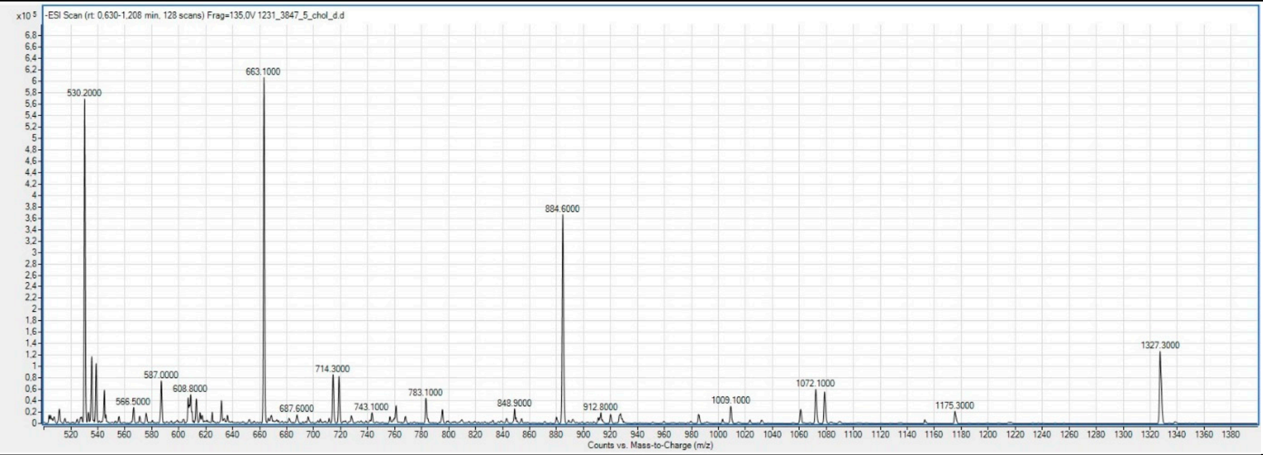

[H5] / 530.2	2656.0 →	M 2656.0
[H4] / 663.1	2656.4 →	M 2656.4
[H3] / 884.6	2656.8 →	M 2656.8
[H2] / 1327.3	2656.6 →	M 2656.6
		M$_{found}$ 2656.5

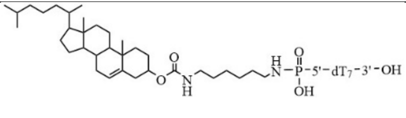

M$_{calcd}$ 2658.2

(3) *Oleyl*-NH-p-d(TTTTTTT)

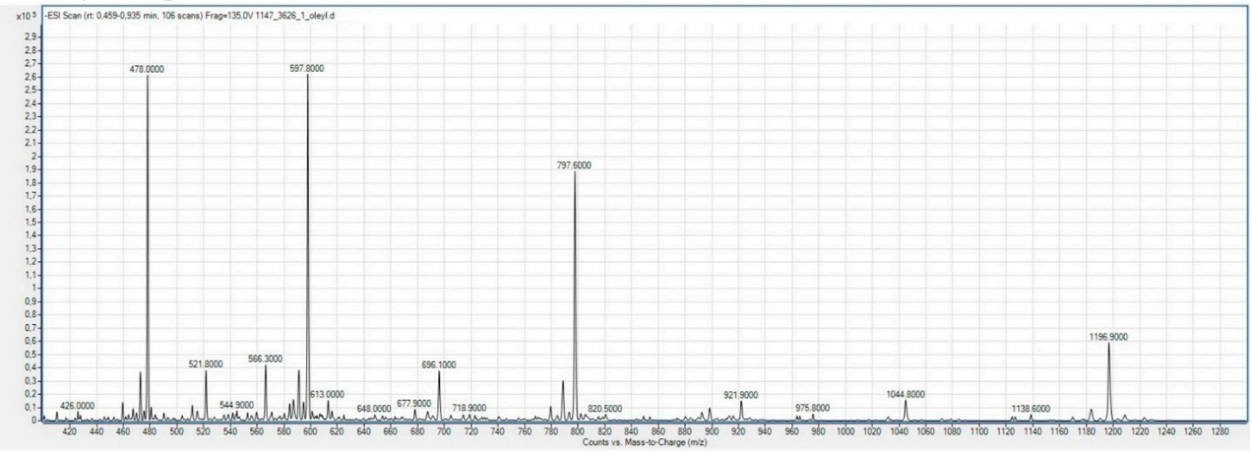

[H5] / 478.0 2395.0 → M 2395.0
[H4] / 597.8 2395.2 → M 2395.2
[H3] / 797.6 2395.8 → M 2395.8
[H2] / 1196.9 2395.8 → M 2395.8
M_{found} 2395.5

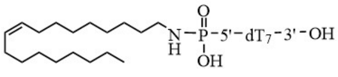

M_{calcd} 2396.8

(4) CH≡C-CH_2-NH-p-d(TTTTTTT)

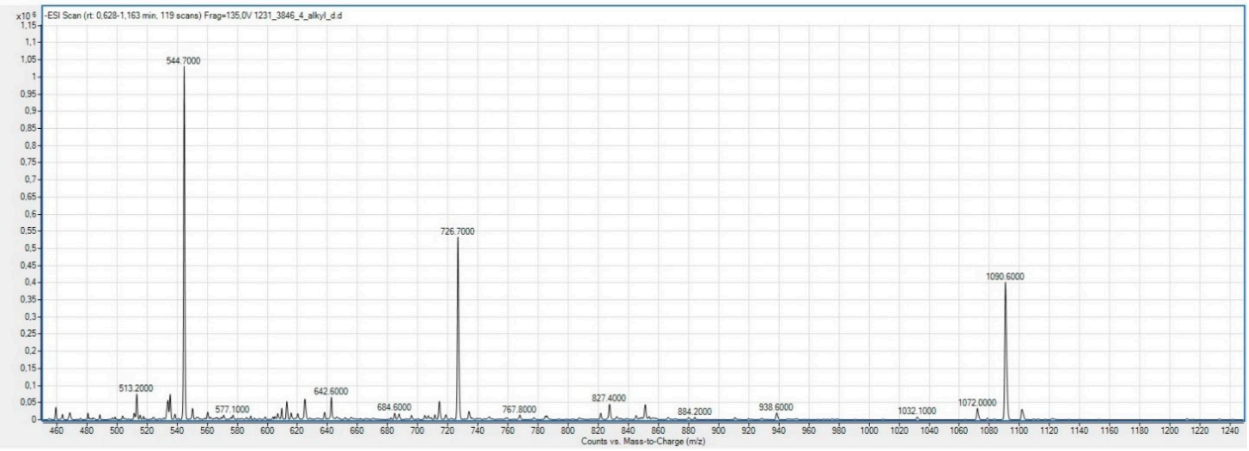

[H4] / 544.7 2182.8 → M 2182.8
[H3] / 726.7 2183.1 → M 2183.1
[H2] / 1090.6 2183.2 → M 2183.2
M_{found} 2183.0

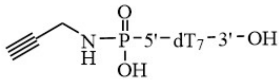

M_{calcd} 2184.4

(5) *Pyr*-CH_2-NH-p-d(TTTTTTT)

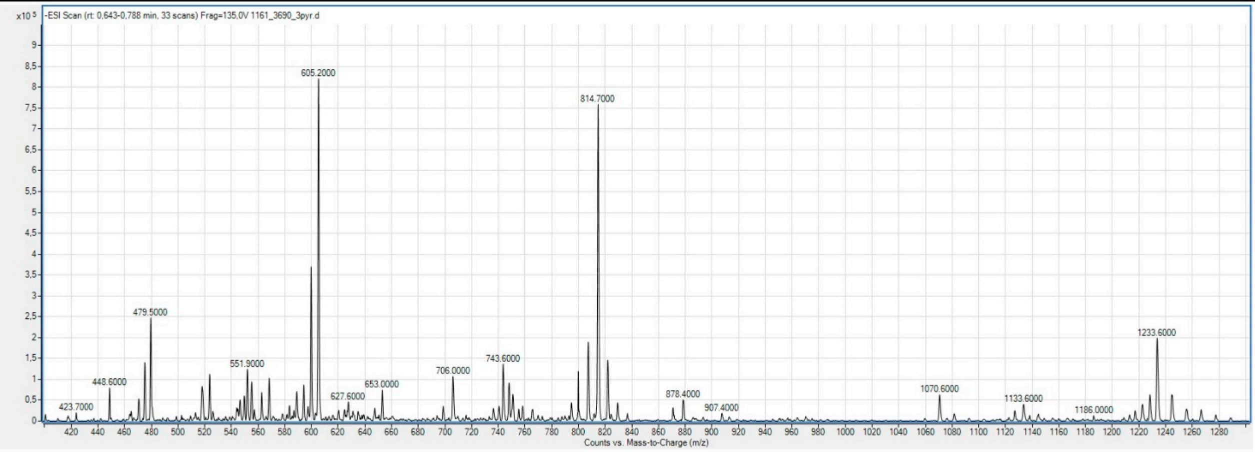

[H5] / 479.5 2402.5 – 2*23(M_{Na+}) + 2*1(M_{H+}) → M 2358.5
[H4] / 605.2 2424.8 – 3*23(M_{Na+}) + 3*1(M_{H+}) → M 2358.8
[H3] / 814.7 2447.1 – 4*23(M_{Na+}) + 4*1(M_{H+}) → M 2359.1
[H2] / 1233.6 2469.2 – 5*23(M_{Na+}) + 5*1(M_{H+}) → M 2359.2

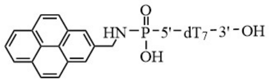

M_{found} 2358.9	M_{calcd} 2360.3

(6) HO-$(CH_2)_3$-NH-p-d(TTTTTTT)

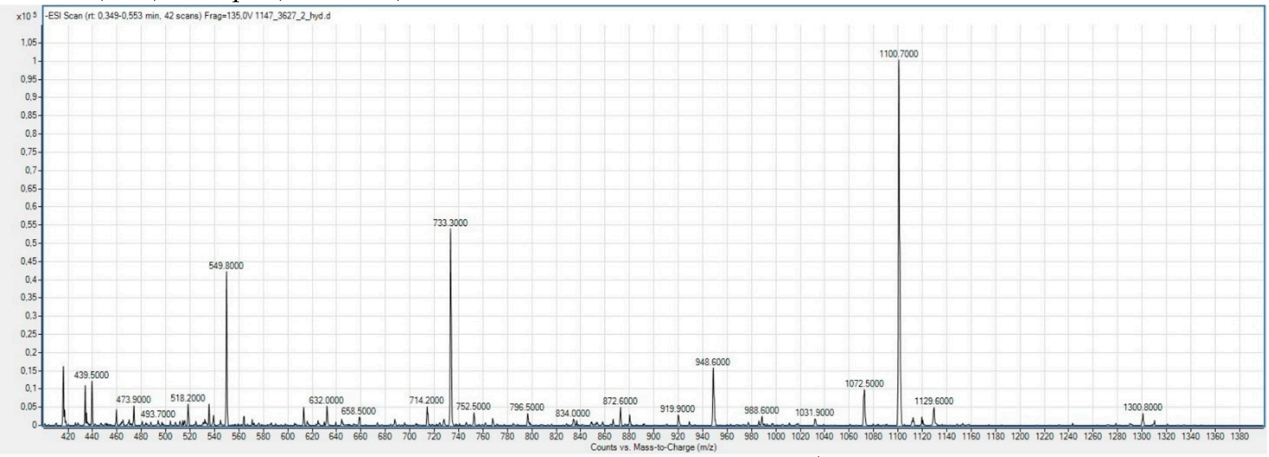

[H4] / 549.8	2203.2 →	M 2203.2	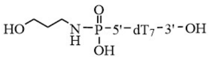
[H3] / 733.3	2202.9 →	M 2202.9	
[H2] / 1100.7	2203.4 →	M 2203.4	
		M_{found} 2203.2	M_{calcd} 2204.4

(7) NH_2-$(CH_2)_6$-NH-p-d(TTTTTTT)

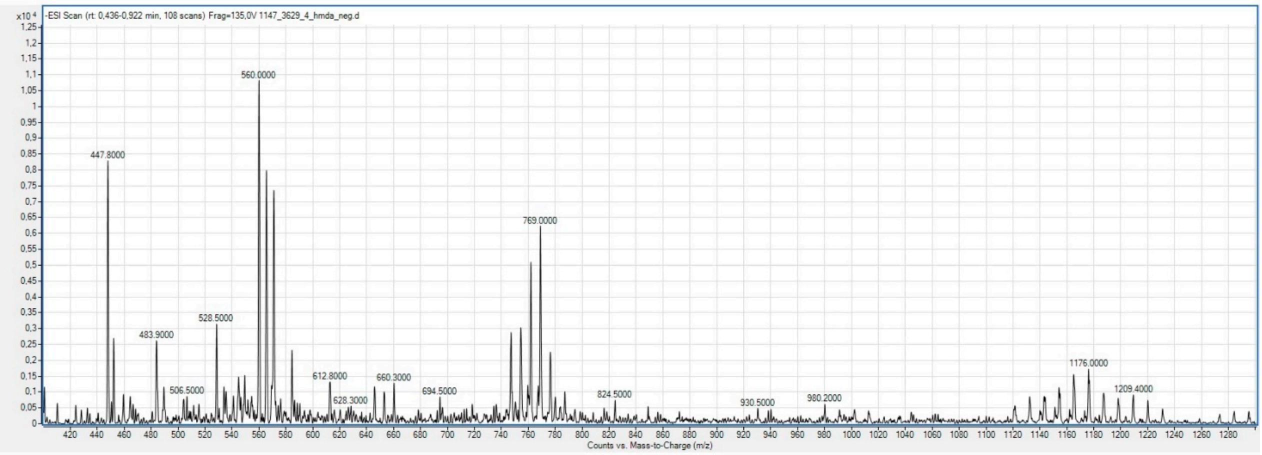

[H5] / 447.8	2244.0 →	M 2244.0	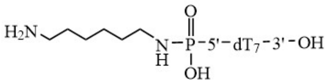
[H4] / 560.0	2244.0 →	M 2244.0	
[H3] / 769.0	2310.0 – 3*23(M_{Na+}) + 3*1(M_{H+}) →	M 2244.0	
[H2] / 1176.0	2354.0 – 5*23(M_{Na+}) + 5*1(M_{H+}) →	M 2244.0	
		M_{found} 2244.0	M_{calcd} 2245.6

(8) *Biot*-NH-$(CH_2)_6$-NH-p-d(TTTTTTT)

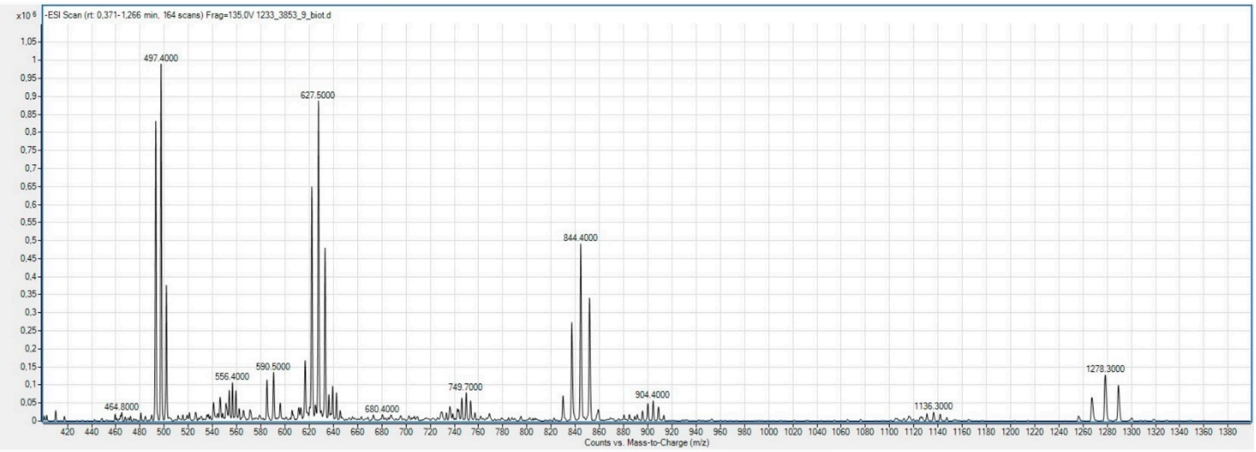

[H5] / 497.4	2492.0 – 1*23(M_{Na+}) + 1*1(M_{H+}) →	M 2470.0	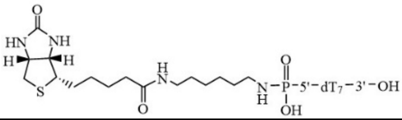
[H4] / 627.5	2514.0 – 2*23(M_{Na+}) + 2*1(M_{H+}) →	M 2470.0	
[H3] / 844.4	2536.2 – 3*23(M_{Na+}) + 3*1(M_{H+}) →	M 2470.2	
[H2] / 1278.3	2558.6 – 4*23(M_{Na+}) + 4*1(M_{H+}) →	M 2470.6	

M_{found} 2470.2 | M_{calcd} 2471.6

(9) CH_3-NH-p-d(TTTTTTT)

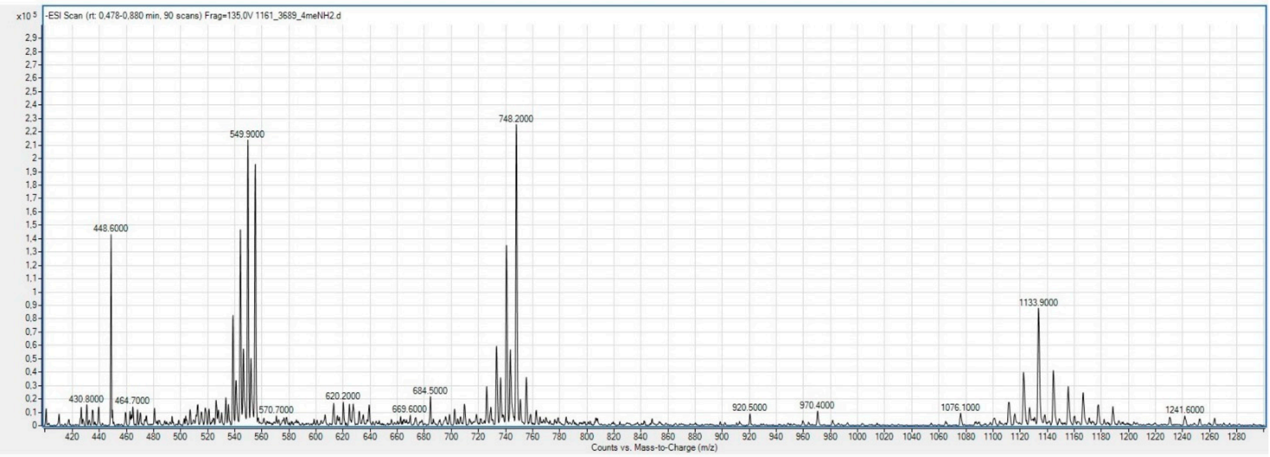

[H4] / 549.9 2203.6 – 2*23(M_{Na+}) + 2*1(M_{H+}) → M 2159.6

[H3] / 748.2 2247.6 – 4*23(M_{Na+}) + 4*1(M_{H+}) → M 2159.6

[H2] / 1133.9 2269.8 – 5*23(M_{Na+}) + 5*1(M_{H+}) → M 2159.8

M_{found} 2159.7

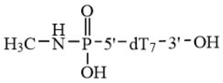

M_{calcd} 2161.1

(10) *Oleyl*-NH-p-$G^mG^mC^mU^mU^mG^mA^mC^mA^m$

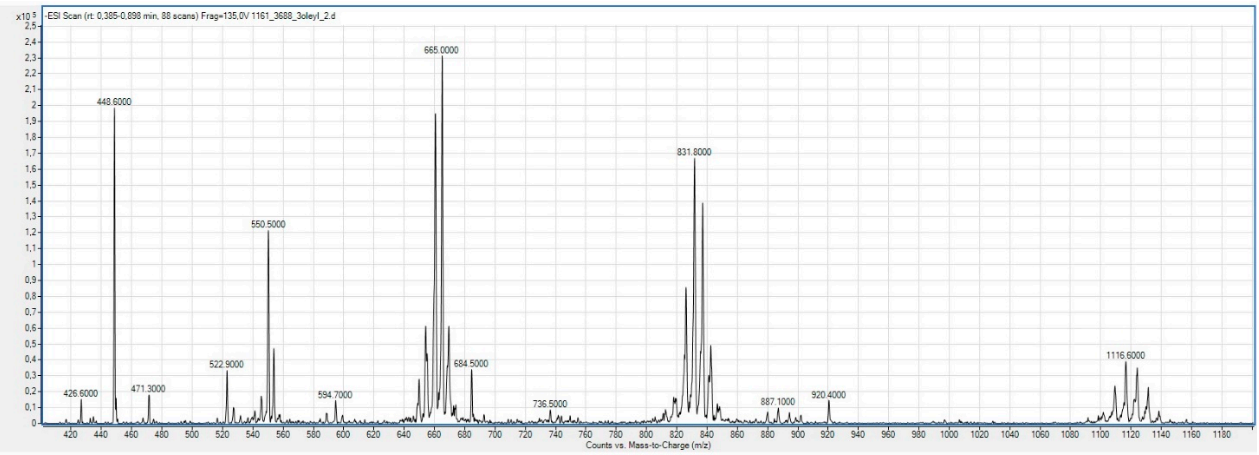

[H6] / 550.5 3309.0 → M 3309.0

[H5] / 665.0 3352.8 – 1*23(M_{Na+}) + 1*1(M_{H+}) → M 3308.8

[H4] / 831.8 3331.2 – 1*23(M_{Na+}) + 1*1(M_{H+}) → M 3309.2

[H3] / 1116.6 3352.8 – 2*23(M_{Na+}) + 2*1(M_{H+}) → M 3308.8

M_{found} 3309.0

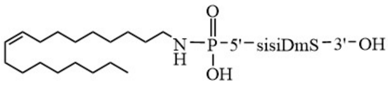

M_{calcd} 3310.3

(11) *MB*-**L₆**-NH-p-$G^mG^mC^mU^mU^mG^mA^mC^mA^m$

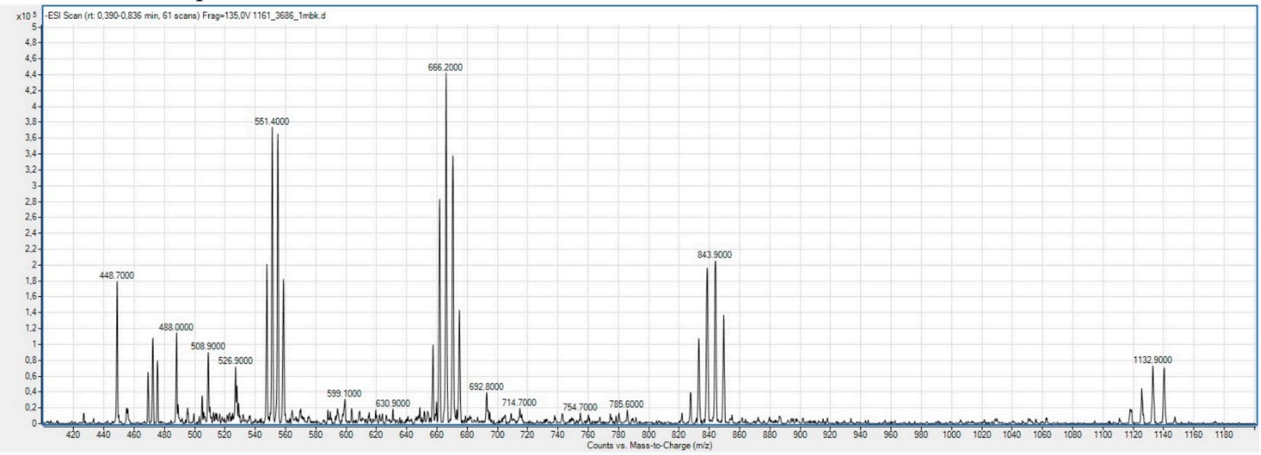

[H6] / 551.4 3314.4 – 1*23(M_{Na+}) + 1*1(M_{H+}) → M 3292.4

[H5] / 666.2 3336.0 – 2*23(M_{Na+}) + 2*1(M_{H+}) → M 3292.0

[H4] / 843.9 3379.6 – 4*23(M_{Na+}) + 4*1(M_{H+}) → M 3291.6

[H3] / 1132.9 3401.7 – 5*23(M_{Na+}) + 5*1(M_{H+}) → M 3291.7

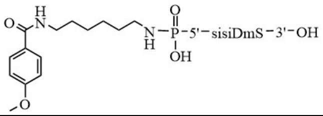

M_{found} 3291.9 | M_{calcd} 3293.3

(12) CH≡C-CH_2-NH-p-$G^mG^mC^mU^mU^mG^mA^mC^mA^m$

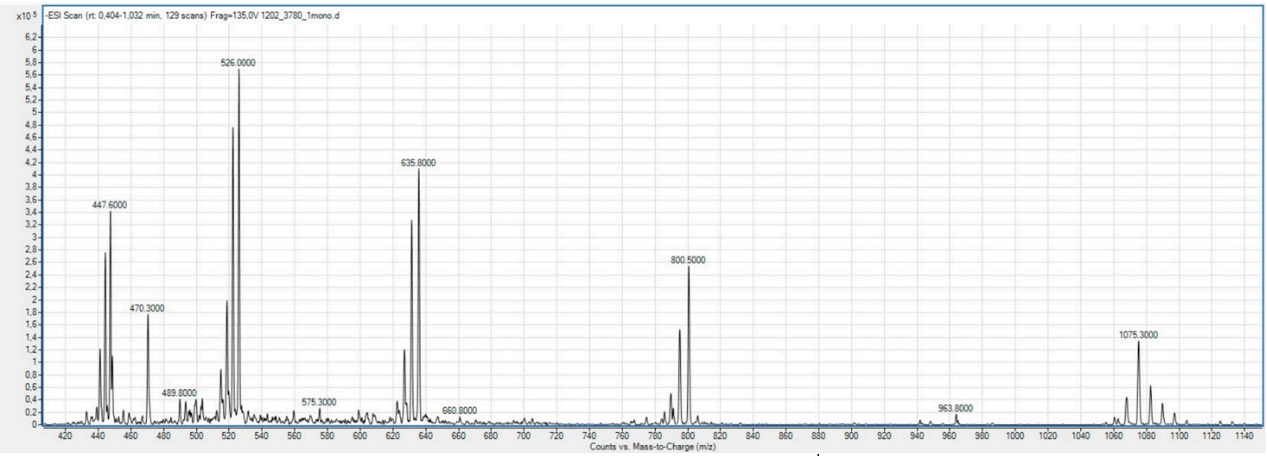

[H7] / 447.6 3140.2 – 2*23(M_{Na+}) + 2*1(M_{H+}) → M 3096.2
[H6] / 526.0 3162.0 – 3*23(M_{Na+}) + 3*1(M_{H+}) → M 3096.0
[H5] / 635.8 3184.0 – 4*23(M_{Na+}) + 4*1(M_{H+}) → M 3096.0
[H4] / 800.5 3206.0 – 5*23(M_{Na+}) + 5*1(M_{H+}) → M 3096.0
[H3] / 1075.3 3228.9 – 6*23(M_{Na+}) + 6*1(M_{H+}) → M 3096.9
M_{found} 3096.2

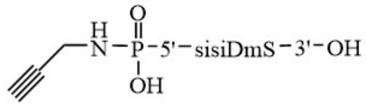

M_{calcd} 3098.1

(13) *FAM-click*-CH_2-NH-p-$G^mG^mC^mU^mU^mG^mA^mC^mA^m$

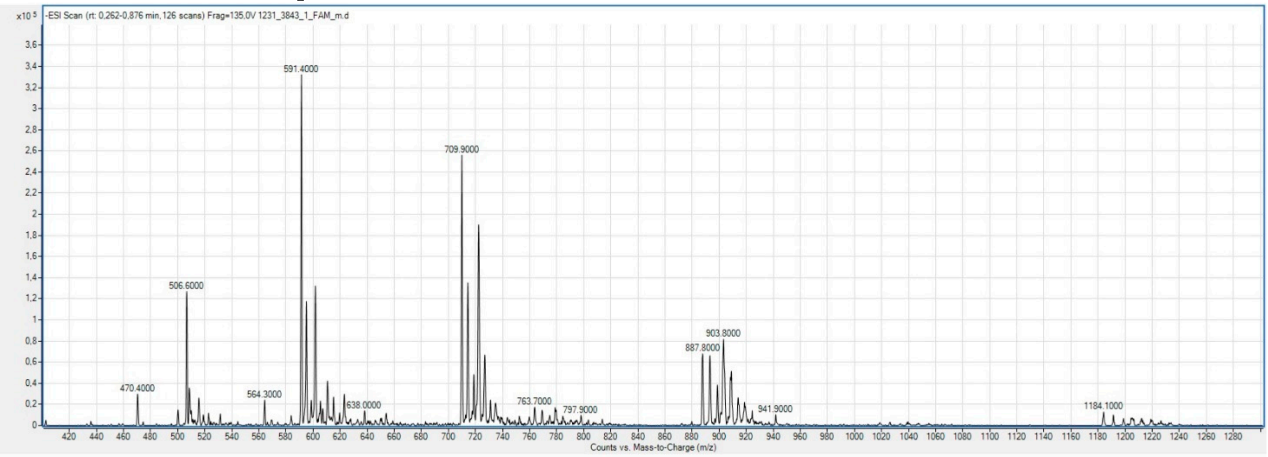

[H7] / 506.6 3553.2 → M 3553.2
[H6] / 591.4 3554.4 → M 3554.4
[H5] / 709.9 3554.5 → M 3554.5
[H4] / 887.8 3555.2 → M 3555.2
[H3] / 1184.1 3555.2 → M 3555.3
M_{found} 3554.5

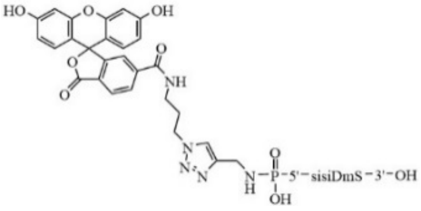

M_{calcd} 3555.5

(14) *MB*-$\mathbf{L}_6$-NH-p-GGCUUGACAAGUUGUAUAUGG^m

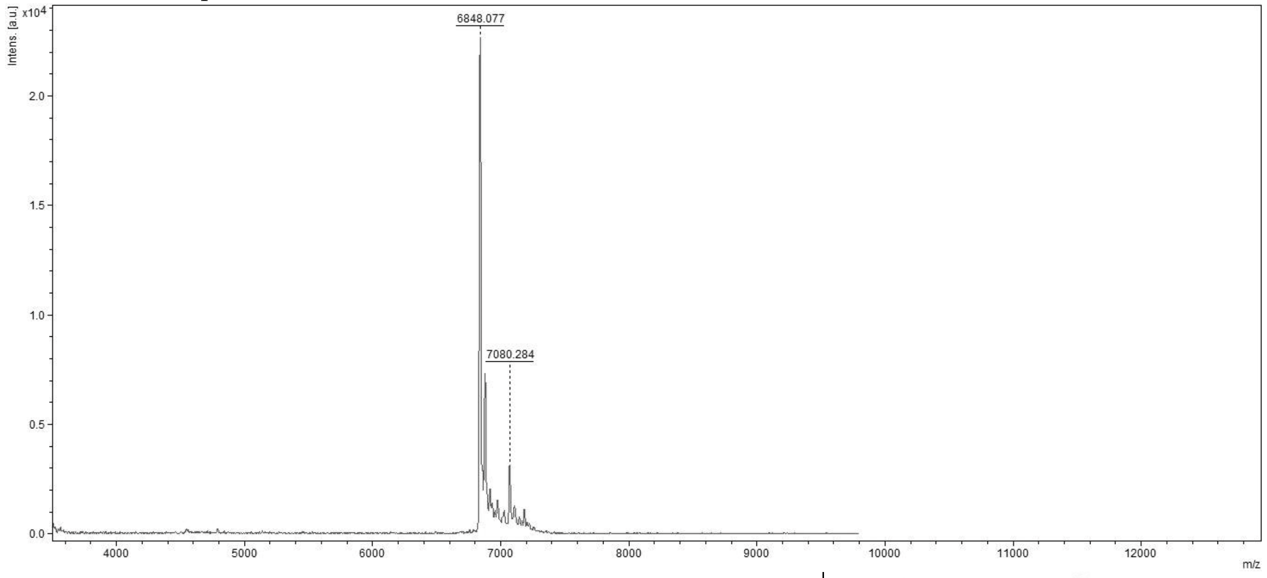

5′-phosphorylated oligonucleotide

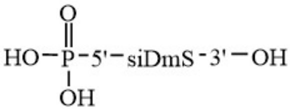

M_{calcd} 6848.0
M_{found} 6848.08

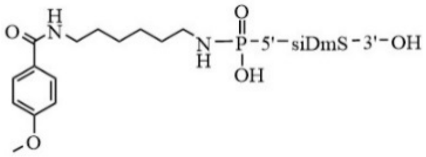

M_{calcd} 7080.4
M_{found} 7080.28

(15) *Chol*-C(O)-$\mathbf{L}_6$-NH-p-GGCUUGACAAGUUGUAUAUGG^m

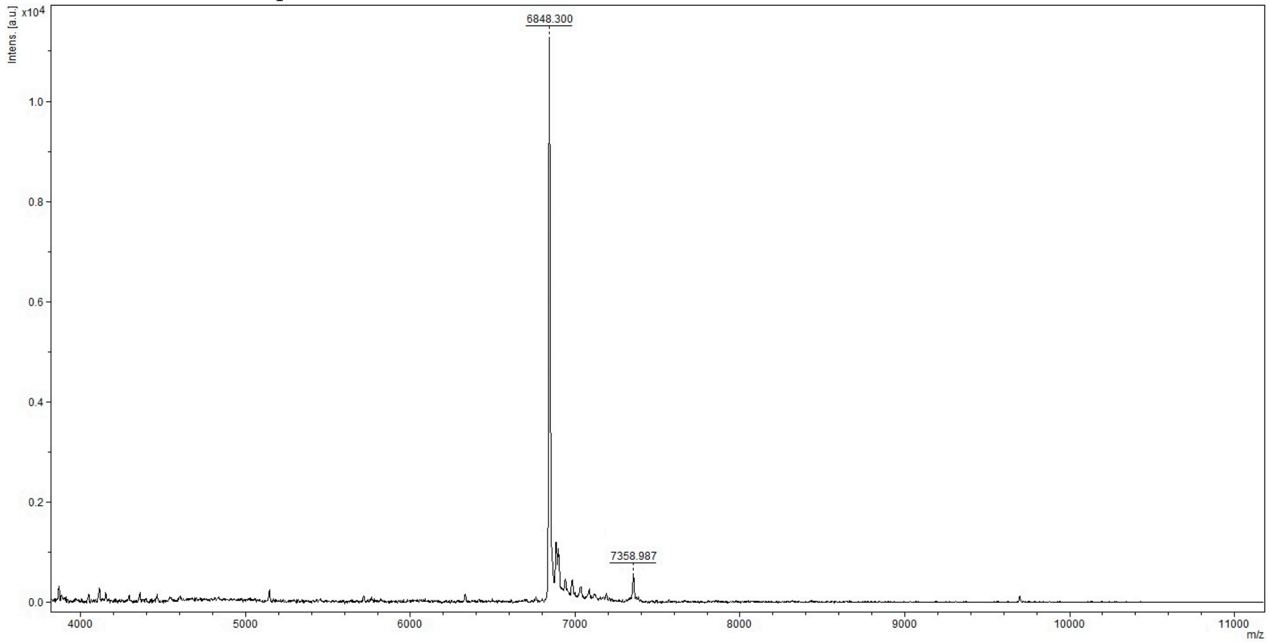

5′-phosphorylated oligonucleotide

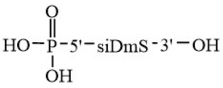

M_{calcd} 6848.0
M_{found} 6848.3

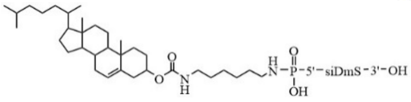

M_{calcd} 7358.9
M_{found} 7358.98

(16) *Oleyl*-NH-p-GGCUUGACAAGUUGUAUAUGG^{m}

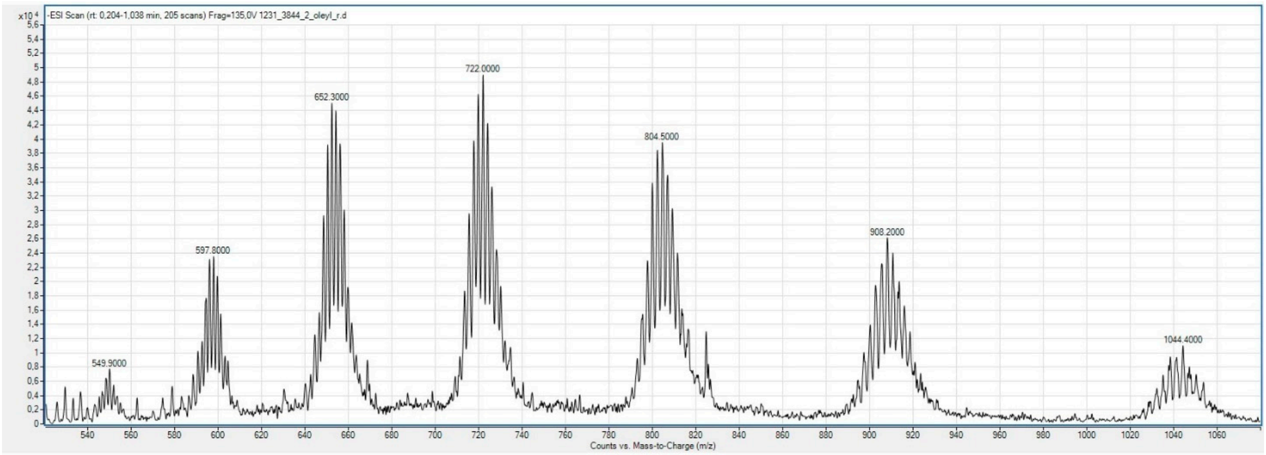

[H13] / 549.9	7161.7 – 3*23(M_{Na+}) + 3*1(M_{H+}) →	M 7095.7
[H12] / 597.8	7185.6 – 4*23(M_{Na+}) + 4*1(M_{H+}) →	M 7097.6
[H11] / 652.3	7186.3 – 4*23(M_{Na+}) + 4*1(M_{H+}) →	M 7098.3
[H10] / 722.0	7230.0 – 6*23(M_{Na+}) + 6*1(M_{H+}) →	M 7098.0
[H9] / 804.5	7249.5 – 7*23(M_{Na+}) + 7*1(M_{H+}) →	M 7095.5
[H8] / 908.2	7272.6 – 8*23(M_{Na+}) + 8*1(M_{H+}) →	M 7097.6
[H7] / 1044.4	7317.8 – 10*23(M_{Na+}) + 10*1(M_{H+}) →	M 7097.8
		M_{found} 7097.2

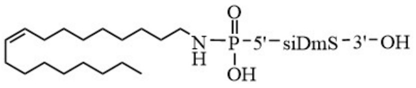

M_{calcd} 7097.6

(17) CH≡C-CH$_2$-NH-p-GGCUUGACAAGUUGUAUAUGG^{m}

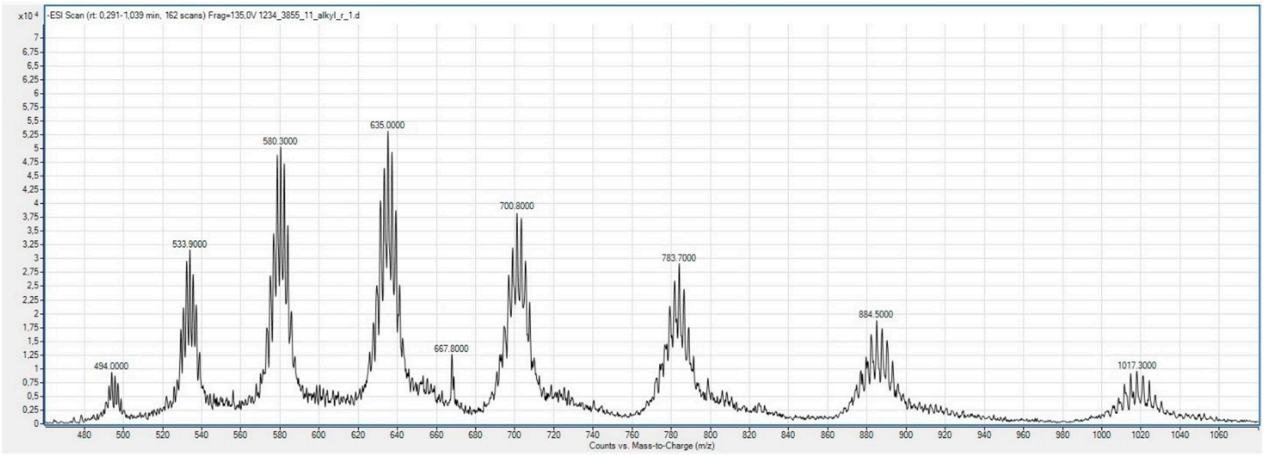

[H14] / 494.0	6930.0 – 2*23(M_{Na+}) + 2*1(M_{H+}) →	M 6886.0
[H13] / 533.9	6953.7 – 3*23(M_{Na+}) + 3*1(M_{H+}) →	M 6887.7
[H12] / 580.3	6975.6 – 4*23(M_{Na+}) + 4*1(M_{H+}) →	M 6887.6
[H11] / 635.0	6996.0 – 5*23(M_{Na+}) + 5*1(M_{H+}) →	M 6886.0
[H10] / 700.8	7018.0 – 6*23(M_{Na+}) + 6*1(M_{H+}) →	M 6886.0
[H9] / 783.7	7062.3 – 8*23(M_{Na+}) + 8*1(M_{H+}) →	M 6886.3
[H8] / 884.5	7084.0 – 9*23(M_{Na+}) + 9*1(M_{H+}) →	M 6886.0
[H7] / 1017.3	7128.1 – 11*23(M_{Na+}) + 11*1(M_{H+}) →	M 6886.1
		M_{found} 6886.4

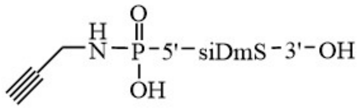

M_{calcd} 6885.1

(18) *GalNAc-click*-CH_2-NH-p-GGCUUGACAAGUUGUAUAUGG^m

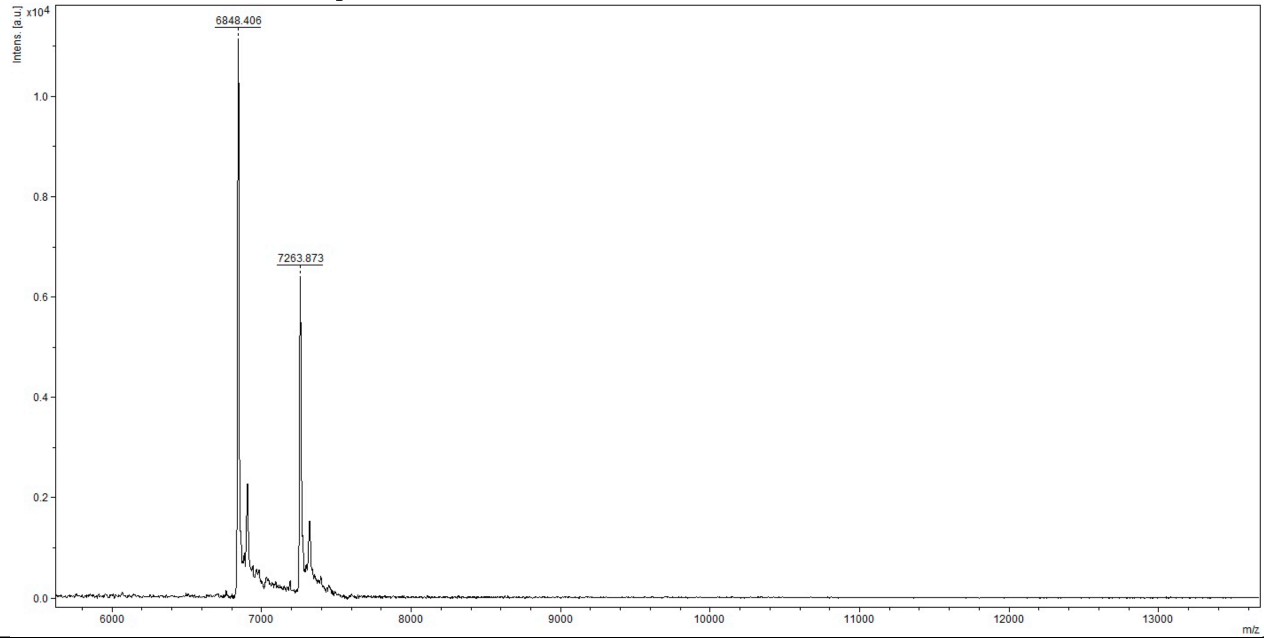

5′-phosphorylated oligonucleotide

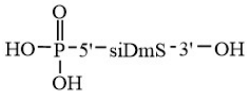

M_{calcd} 6848.0
M_{found} 6848.4

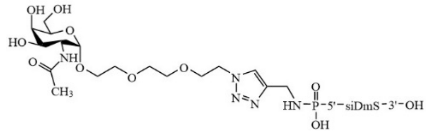

M_{calcd} 7263.7
M_{found} 7263.87

Chol-C(O)-**L**$_6$-NH–, Cholesteryl-6-aminohexylcarbamate residue; *Oleyl*-NH–, oleylamine residue; *Pyr*-CH_2-NH–, pyrenemethylamine residue; *MB*-**L**$_6$-NH-p-, *N*-(6-aminohexyl)-4-methoxybenzamide residue; NH_2-$(CH_2)_6$-NH-, 1,6-diaminohexane residue; HO-$(CH_2)_3$-NH-, 3-amino-1-propanole residue; CH≡C-CH_2-NH–, propargylamine residue; *Biot*-, Biotin residue (see aslo Figure S2); *FAM-click*-CH_2-NH-, FAM residue with 1,2,3-triazole linker (see also Figure S3); *GalNAc-click*, GalNAc residue with 1,2,3-triazole linker (see also Figure S3); -p-, -P(O)(OH)-; **L**$_6$ -, -NH$(CH_2)_6$-; N, ribonucleotide; N^m, 2′-O-methylribonucleotide; d(N), deoxyribonucleotide.

Table S3. ^{1}H-NMR spectra of amino containing ligand.

(A) ^{1}H-NMR spectrum of cholesteryl-6-aminohexylcarbamate (**I**). NMR spectrum was measured with $CDCl_3$ as a solvent using AVANCE III 300 NMR spectrometer. The assignment of peaks in the NMR spectrum is given in the Materials and Methods.

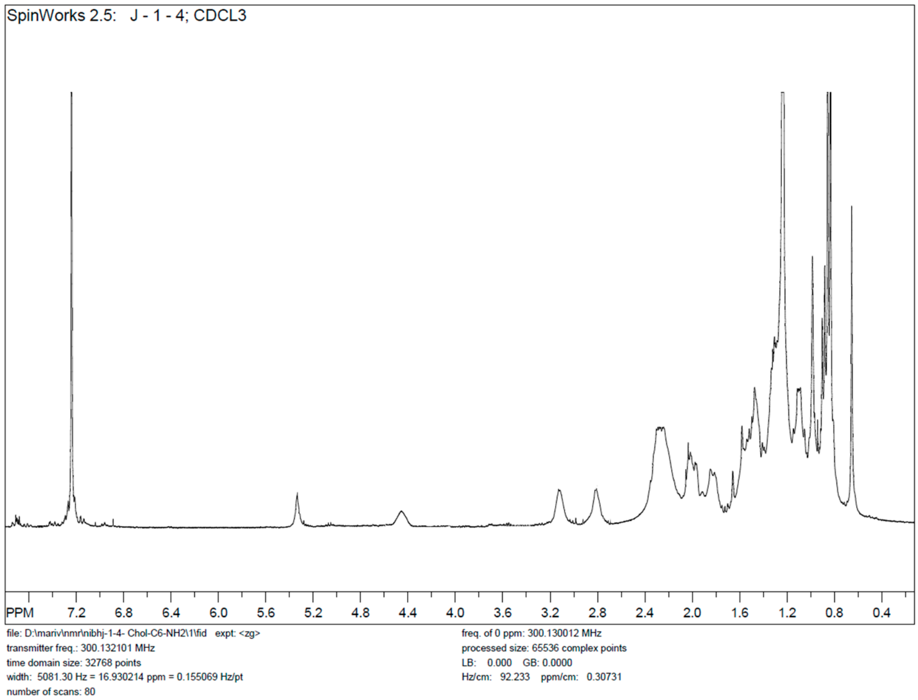

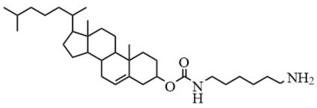

(B) ^{1}H-NMR spectrum of *N*-Boc-protected *N*-Boc-(6-aminohexyl)-4-methoxybenzamide. NMR spectrum was measured with $CDCl_3$ as a solvent using AVANCE III 400 NMR spectrometer. The assignment of peaks in the NMR spectrum is given in the Materials and Methods.

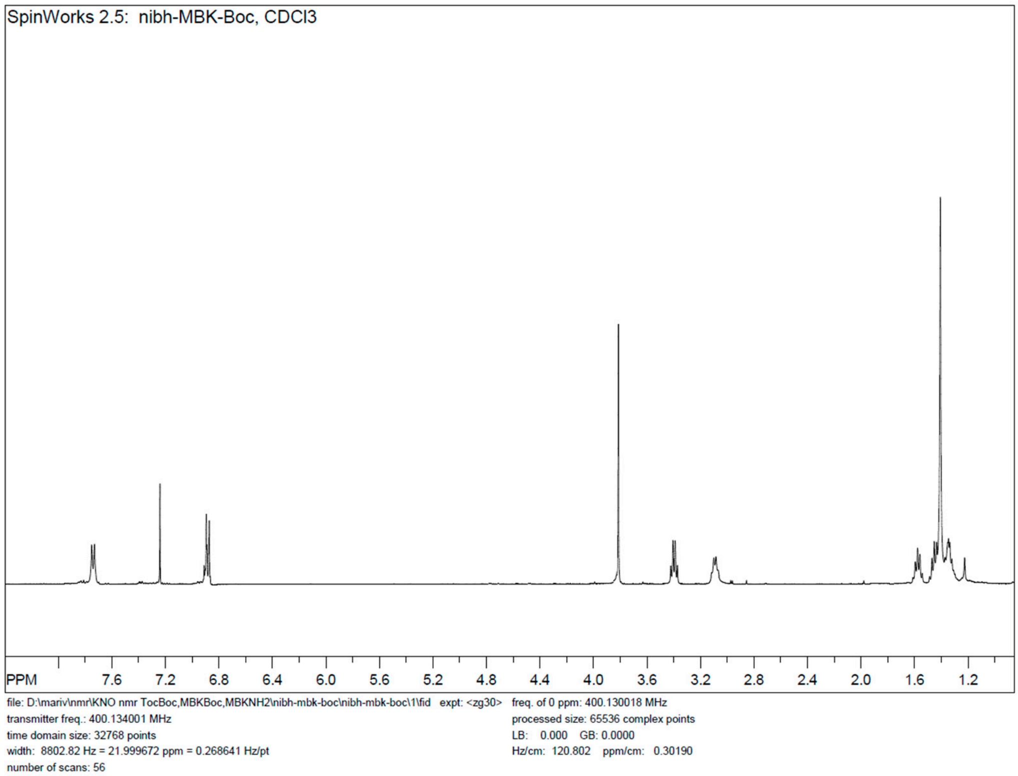

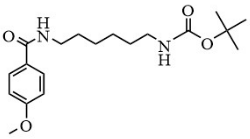

(C) ^{1}H-NMR spectrum of *N*-(6-aminohexyl)-4-methoxybenzamide (**II**). NMR spectrum was measured with $CDCl_3$ as a solvent using AVANCE III 400 NMR spectrometer. The assignment of peaks in the NMR spectrum is given in the Materials and Methods.

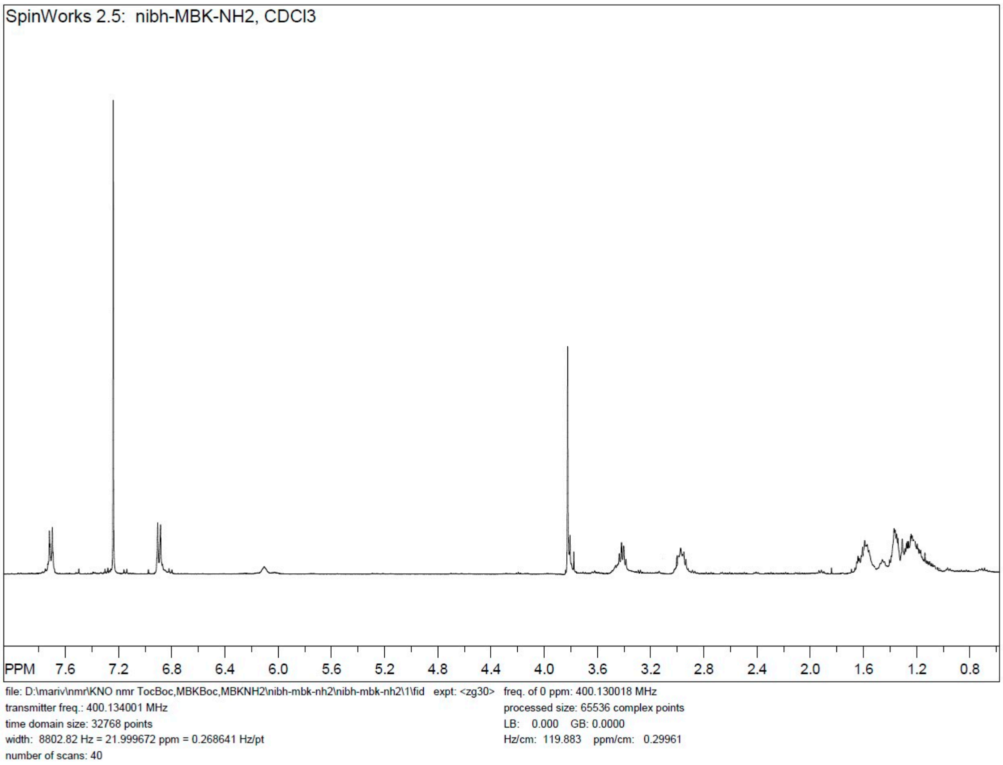
SpinWorks 2.5: nibh-MBK-NH2, CDCl3
PPM 7.6 7.2 6.8 6.4 6.0 5.6 5.2 4.8 4.4 4.0 3.6 3.2 2.8 2.4 2.0 1.6 1.2 0.8
file: D:\mariv\nmr\KNO nmr TocBoc,MBKBoc,MBKNH2\nibh-mbk-nh2\nibh-mbk-nh2\1\fid expt: <zg30>
transmitter freq.: 400.134001 MHz
time domain size: 32768 points
width: 8802.82 Hz = 21.999672 ppm = 0.268641 Hz/pt
number of scans: 40
freq. of 0 ppm: 400.130018 MHz
processed size: 65536 complex points
LB: 0.000 GB: 0.0000
Hz/cm: 119.883 ppm/cm: 0.29961

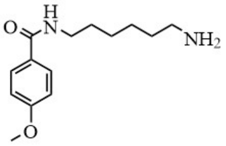
O
H
N
NH2
O

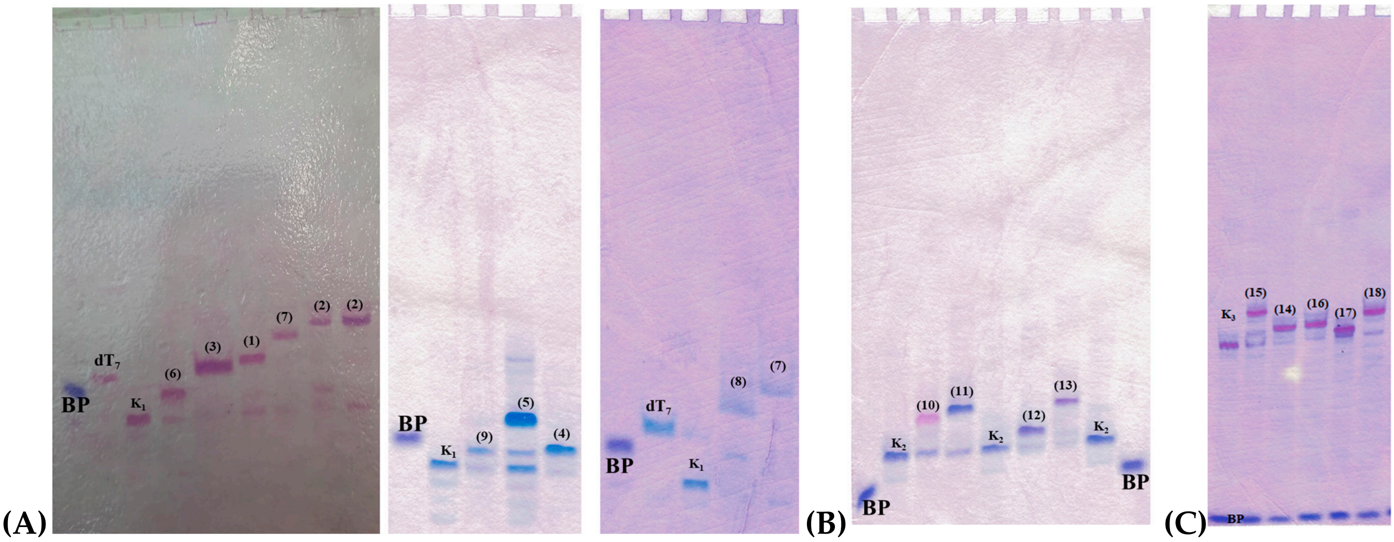

Figure S4. Full-size images of electropherograms after PAGE analysis and Stains-all staining for 5'-phosphorylated oligonucleotides and their conjugates (1-18).**(A)** 5'-p-dT_7 (K_1) and conjugates (**1**-**9**); **(B)** 5'-p-sisiDmS (K_2*)* and conjugates (**10**-**13**); **(C)** 5'-p-siDmS (K_3) and conjugates (**14**-**18**). Conditions: 15% denaturating PAAG (7M urea, acrylamide/*N*,*N*'-methylene bis-acrylamide (19/1)) in TBE buffer. Gel stained with "Stains-all". BP – bromophenol blue.

Experimental Section S1. Automated synthesis of polymer-bound oligonucleotides

Oligodeoxyribonucleotides,oligo(2′-O-methylribonucleotides), oligoribonucleotides and their 5′-phosphate derivatives were synthesized on an automatic ASM-800 synthesizer at 0.4 mmol scale using solid-phase phosphoramidite synthesis protocols optimized for the instrument, with a 3 min coupling step for deoxy phosphoramidites (0.05 M in CH_3CN), 10 min coupling step for 2′-O-TBDMS protected and CPR phosphoramidites (0.1 M in CH_3CN), 6 min coupling step for 2′-O-methyl phosphoramidites (0.05 M in CH_3CN) and 5-ethylthio-1H-tetrazole (0.25 M in CH_3CN) as an activating agent. A mixture of propionic anhydride (10%, v/v) with 2,6-lutidine (10%, v/v) in THF and N-methylimidazole (16%, v/v) in THF were utilized as capping reagents. The oxidizing agent was 0.02 M iodine in pyridine/water/THF (1/9/90, v/v/v). Dichloroacetic acid (3%, v/v) in CH_2Cl_2 was used as detritylating reagent.

Table S4. Stability of the P-N-bond within the oligonucleotide conjugates (14-16, 18) at different pH values.

**(14)** *MB*-**L**$_6$-NH-p-GGCUUGACAAGUUGUAUAUGG^m	
pH 4.5	(**14**) 1h 2h 4h 6h 24h **(*)** 1h 2h 4h 6h 24h (**14**) 1h 2h 4h 6h 24h **(*)**<br>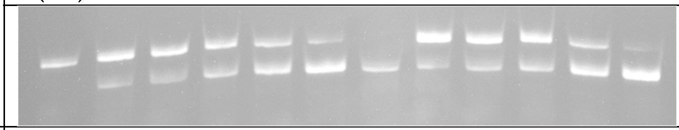 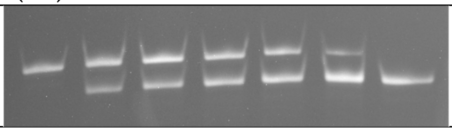
pH 5.2	**(*)** 1h 2h 4h 6h 24h 1h 2h 4h 6h 24h (**14**) **(*)** 1h 2h 4h 6h 24h (**14**)<br>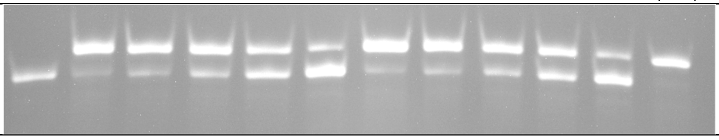 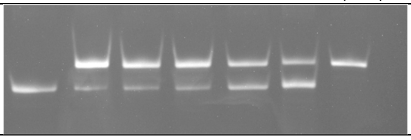
pH 6.0	(**14**) 1h 2h 4h 6h 24h 1h 2h 4h 6h 24h **(*)** (**14**) 1h 2h 4h 6h 24h **(*)**<br>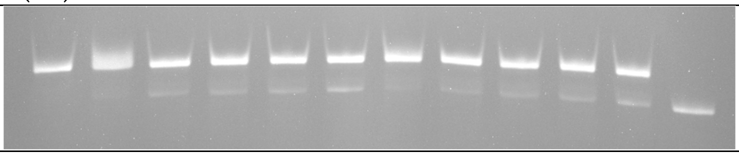 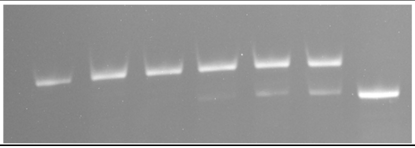

**(15)** *Chol*-C(O)-**L**$_6$-NH-p-GGCUUGACAAGUUGUAUAUGG^m	
pH 4.5	(**15**) 1h 2h 4h 6h 24h 1h 2h 4h 6h 24h **(*)** (**15**) **(*)** 1h 2h 4h 6h 24h<br>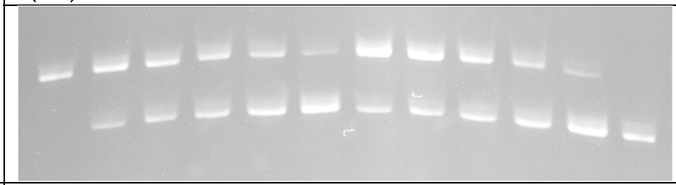 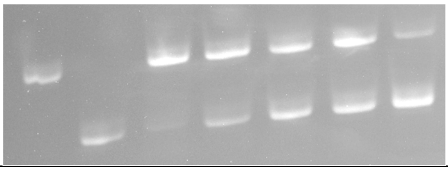
pH 5.2	(**15**) 1h 2h 4h 6h 24h 1h 2h 4h 6h 24h **(*)** 1h 2h 4h 6h 24h (**15**)<br>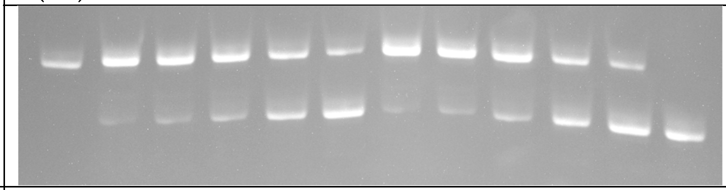 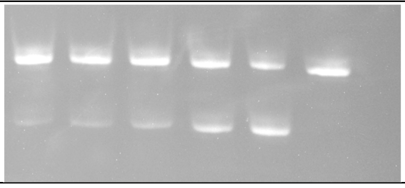
pH 6.0	**(*)** 1h 2h 4h 6h 24h (**15**) 1h 2h 4h 6h 24h **(*)** 1h 2h 4h 6h 24h **(*)** (**15**)<br>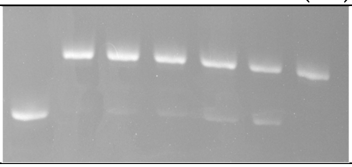 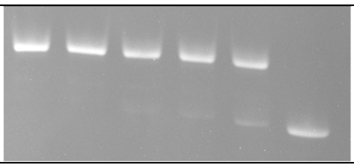 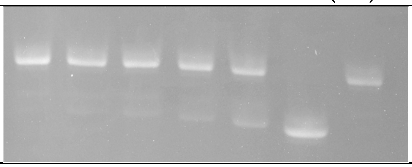

**(16)** *Oleyl*-NH-p-GGCUUGACAAGUUGUAUAUGG^{m}	
pH 4.5	(**16**) 1h 2h 4h 6h 24h 1h 2h 4h 6h 24h (**16**) (**16**) (*****) 1h 2h 4h 6h 24h 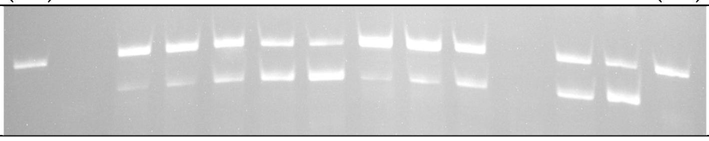 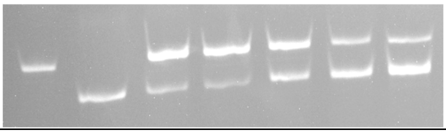
pH 5.2	(**16**) 1h 2h 4h 6h 24h 1h 2h 4h 6h 24h (*****) 1h 2h 4h 6h 24h (**16**) 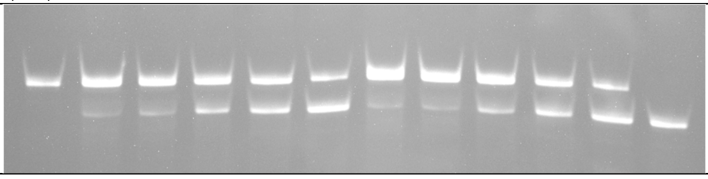 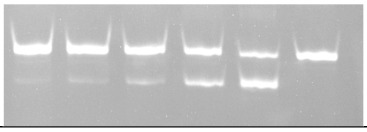
pH 6.0	(*****) 1h 2h 4h 6h 24h (**16**) (*****) (**16**) 1h 2h 4h 6h 24h 1h 2h 4h 6h 24h (**16**) (*****) 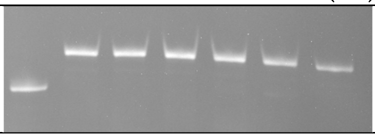 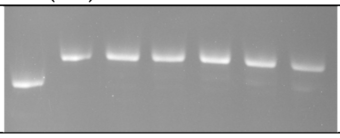 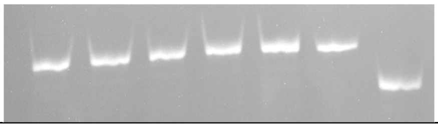

**(18)** *GalNAc-click*-CH_2-NH-p-GGCUUGACAAGUUGUAUAUGG^{m}	
pH 4.5	(**18**) 1h 2h 4h 6h 24h 1h 2h 4h 6h 24h (**18**) (**18**) 1h 2h 4h 6h 24h (*****) 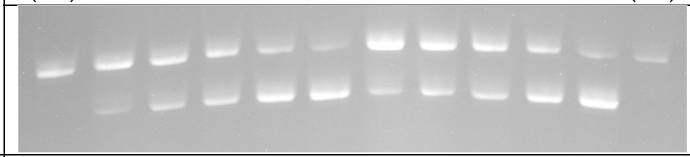 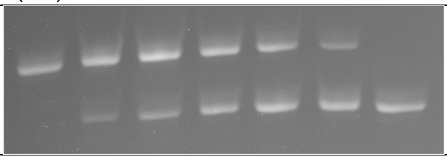
pH 5.2	(**18**) 24h 6h 4h 2h 1h (*****) 24h 6h 4h 2h 1h (*****) 1h 2h 4h 6h 24h (**18**) 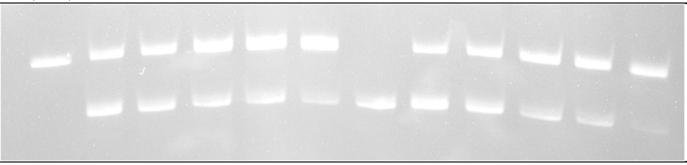 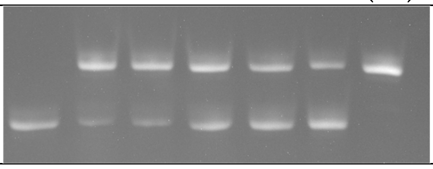
pH 6.0	(*****) 1h 2h 4h 6h 24h 1h 2h 4h 6h 24h (**18**) 1h 2h 4h 6h 24h (**18**)(*****) 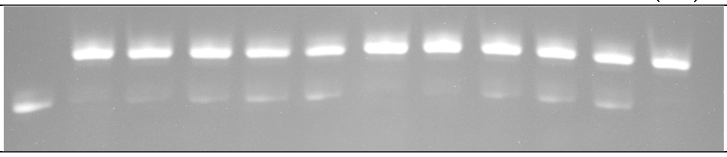 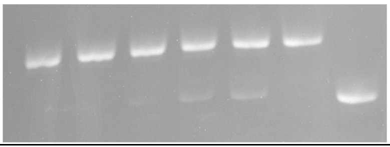

(*) - 5′-phosphorylated oligonucleotide 5'-p-siDmS (5′-p-GGCUUGACAAGUUGUAUAUGG^{m}). *MB*-**L**$_6$-NH-p-, *N*-(6-aminohexyl)-4-methoxybenzamide residue; *Chol*-C(O)-**L**$_6$-NH–, cholesteryl-6-aminohexylcarbamate residue; *Oleyl*-NH–, oleylamine residue; *GalNAc-click*, GalNAc residue with 1,2,3-triazole linker; -p-, -P(O)(OH)-; **L**$_6$ -, -NH$(CH_2)_6$-; N, ribonucleotide; N^{m}, 2′-O-methylribonucleotide.Conditions: 15% denaturating PAAG (7M urea, acrylamide/*N,N'*-methylene bis-acrylamide (19/1)) in TBE buffer. Gel stained with ethidium bromide.
